# Innate lymphoid cells in the spotlight: from biomarkers to blueprint for innovative immunotherapy

**DOI:** 10.3389/fimmu.2025.1655730

**Published:** 2025-09-02

**Authors:** I-Chih Kuo, Julyanne Brassard, Peter W. Zandstra, Kelly M. McNagny

**Affiliations:** ^1^ School of Biomedical Engineering, University of British Columbia, Vancouver, BC, Canada; ^2^ Department of Experimental Medicine, University of British Columbia, Vancouver, BC, Canada; ^3^ Michael Smith Laboratories, University of British Columbia, Vancouver, BC, Canada; ^4^ Medical Genetics, University of British Columbia, Vancouver, BC, Canada; ^5^ Centre for Heart Lung Innovation, St. Paul’s Hospital, University of British Columbia, Vancouver, BC, Canada

**Keywords:** innate lymphoid cells (ILCs), cancer, autoimmune diseases, biomarkers, chronic inflammatory diseases, immunotherapy, regenerative medicine

## Abstract

Since their discovery, innate lymphoid cells (ILCs) have emerged as key players in immune regulation, tissue homeostasis, and disease pathogenesis. Early research focused on defining ILC subsets, including ILC1s, ILC2s, ILC3s, and lymphoid tissue inducer (LTi) cells, by distinguishing their development, transcriptional profiles, and effector functions relative to T cells. Subsequent studies characterized the tissue-resident nature of ILCs and mapped their context-dependent phenotypes across diverse organs. In parallel, increasing evidence linked ILC subset imbalances to the pathogenesis of autoimmune diseases and various cancers. Recent work has leveraged circulating ILC frequencies and phenotypes as potential biomarkers for disease severity and progression. Notably, the immunomodulatory, tissue-reparative, and cytotoxic functions of helper ILCs have attracted interest as novel therapeutic avenues. Current strategies to harness ILCs for therapy include *ex vivo* expansion of autologous or allogeneic ILCs, derivation of ILC-like cells from umbilical cord blood or pluripotent stem cells (PSCs), and engineering of ILCs with chimeric antigen receptors (CARs) to enhance antigen specificity. Additionally, cytokine modulation and immune checkpoint blockade are being explored to sustain or redirect ILC function in disease contexts. This review synthesizes recent advances in understanding the functional diversity, plasticity, and tissue residency of ILC subsets, emphasizing their interactions with other immune and stromal cells, and their roles as predictive, diagnostic, and therapeutic targets in autoimmune diseases and cancers. Key translational challenges, including subset heterogeneity, plasticity, tissue-restricted residency, and limited scalability, remain barriers to clinical application. However, emerging multi-omic technologies, single-cell atlases, and synthetic biology approaches are accelerating efforts to map ILC states with unprecedented resolution and guide rational therapeutic design. Looking forward, integration of ILC-based therapies with regenerative medicine, cellular engineering, and immuno-oncology platforms holds promise for developing next-generation precision immunotherapies. By bridging fundamental biology with translational innovation, this field is poised to expand the therapeutic landscape for both autoimmune and malignant diseases.

## Graphical Abstract

Figure content is related to the abstract. Figure created using Biorender.

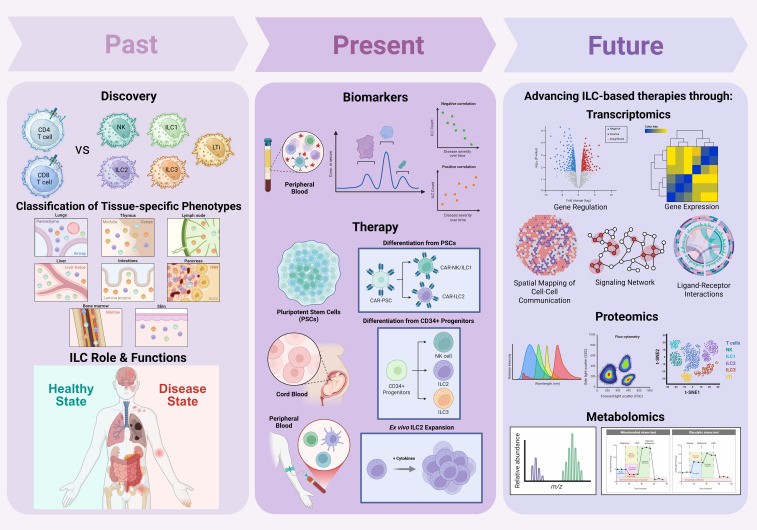

## 1 Introduction

### 1.1 ILC characteristics and functions

Innate lymphoid cells (ILCs) are a family of tissue-resident immune cells that contribute critically to immune homeostasis, barrier integrity, and early defense responses. While they lack cell surface expression of antigen-specific receptors, ILCs/NK cells mirror the effector programs of adaptive CD4^+^ T helper (Th) and CD8^+^ cytotoxic T cells, producing rapid cytokine and effector responses to local environmental cues (1, 2). Unlike T and B cells, ILCs are defined as lineage-negative (Lin^-^), lacking surface markers of T cells (CD3, TCRαβ, TCRγδ), B cells (CD19), myeloid cells (CD14, CD11b, CD11c), mast cells (FcϵRI), and hematopoietic progenitors (CD34) (3, 4). Positive identification of ILCs relies on tissue residency, subset-specific markers and transcription factors. ILCs are broadly classified into five distinct subsets: natural killer (NK) cells, ILC1, ILC2, ILC3, and lymphoid tissue inducer (LTi) cells (5). NK cells are cytotoxic and circulate systemically, whereas helper ILCs (ILC1, ILC2, ILC3 and LTi) are predominantly tissue-resident and coordinate local tissue-specific immunity (6). Subsets are defined by key transcription factors: Eomes and T-bet in NK cells, T-bet in ILC1s, GATA3 and RORα in ILC2s, and RORγt in ILC3s and LTi cells (2) Tissue distribution of ILCs is non-uniform. ILC1s are enriched in liver, skin, and gut; ILC2s predominate in lung and skin; ILC3s are highly abundant in the intestinal lamina propria; and LTi cells localize to secondary lymphoid tissues such as Peyer’s patches and tonsils (5, 7). Most helper ILC subsets (ILC1, ILC2, ILC3) and LTis express CD127 (IL-7Rα) and CD161 (8, 9). NK cells are identified by high expression of CD56 (in humans), NKp46 (NCR1), NKG2D, and CD16, and lack of CD3; they exhibit potent cytotoxic activity via perforin and granzymes (10). Human NK cells can be functionally divided into CD56^high^ and CD56^low^ subsets: CD56^high^ NK cells, enriched in lymphoid tissues and inflamed sites, are potent cytokine producers with immunoregulatory and immunostimulatory roles, while CD56^dim^ NK cells dominate in circulation and serve as primary cytotoxic effectors, contributing to target cell lysis (11, 12). ILC1s express CD49a, and CD69, may variably express NKp46 depending on tissue, and lack Eomes and cytotoxic machinery, thus, distinguishing them from NK cells (2, 9). ILC2s express CRTH2 (CD294), ST2 (IL-33R), CD25, and variably CD117 (c-Kit) (9, 13). ILC3s express CD117 and are further subdivided based on NCR expression: NKp44^+^ ILC3s (humans), NKp46^+^ or NKp46^-^ (mice and humans), and CCR6^+^ ILC3s (2, 14). LTi cells express CCR6, and high levels of LTα1β2, and are indispensable for secondary lymphoid organ development during embryogenesis (14).

ILCs function as cytokine hubs, responding to tissue-derived signals and orchestrating local immune responses. ILC1s primarily produce interferon-gamma (IFN-γ) and tumor necrosis factor (TNF), mediating type 1 immunity against intracellular pathogens, while also contributing to tissue inflammation and damage (9). ILC2s, in contrast, secrete IL-5, IL-13, and amphiregulin, promoting type 2 immune responses to helminths, regulating tissue repair, fibrosis, and maintaining homeostasis (9, 13). ILC3s secrete IL-17 and IL-22, supporting epithelial barrier integrity, anti-bacterial responses, and modulating host–microbiota interactions (2, 14). LTi cells arise early in development and play an indispensable role in secondary lymphoid organ development during embryogenesis and neonatal life. They contribute to mucosal immunity and immune homeostasis through production of IL-17, IL-22, granulocyte-macrophage colony-stimulating factor (GM-CSF), and membrane-bound LTα1β2 (14, 15). In mice, CCR6^+^ ILC3s overlap with LTi cells—a subset not found in adult human peripheral blood, but present in lymphoid tissues (14). Cytokine profiles of ILC3s vary by subtype: in humans, NKp44^-^ ILC3s produce IL-17, whereas NKp44^+^ ILC3s produce IL-22 exclusively (15).

ILCs have been reported to exhibit plasticity, enabling transdifferentiation across subsets in response to local cytokine cues (2, 16). These transitions are not unidirectional but reflect a dynamic spectrum of states influenced by signals and transcriptional programming. For example, IL-1β, IL-12 and IL-18 promote the conversion of ILC2s and ILC3s to ILC1-like cells, driven by upregulation of T-bet and Aiolos, and downregulation of GATA3 or RORγt (2, 16). This plasticity is reinforced by IL-12/IL-18-induced JAK-STAT4 signaling, which activates T-bet and Runx3 expression, promoting chromatin remodeling and IFN production (2). Conversely, IL-23 and IL-1β, in combination with retinoic acid, reinforce ILC3 identity (RORγt^+^ IL-17^+^/IL-22^+^) through STAT3-mediated induction of c-Maf and Batf, two factors that suppress T-bet expression and stabilize RORγt (16, 17). IL-4-induced STAT6 signaling promotes ILC2 maintenance by upregulating GATA3, while Batf and RORα further support type 2 cytokine production and inhibit ILC1-like polarization (2). Bcl11b further reinforces ILC2 fate by repressing alternative lineage genes and sustaining expression of Gfi1, RORα, and GATA3. Beyond these axes, ILC2↔ILC3 transitions are influenced by IL-23, TGF-β, and vitamin D_3_, which modulate RORγt induction and IL-17 production, while NK↔ILC1 plasticity is triggered by IL-12 and TGF-β through T-bet upregulation and Eomes downregulation (15). Altogether, ILC plasticity is governed by an intricate interplay of cytokine signaling, transcription factors, and tissue-derived cues. This dynamic regulation would allow ILCs to adapt to changing tissue environments and fine-tune their effector programs to diverse immune challenges.

Metabolic programming further regulates ILC function. Fatty acid oxidation sustains ILC2s in nutrient-variable barrier tissues, ILC1s and NK cells rely on glycolysis for rapid effector responses, while ILC3s balance oxidative phosphorylation to support barrier maintenance (18, 19). In this context, dysregulated metabolism likely contributes to ILC-driven pathologies such as asthma, fibrosis, and cancer (18).

Strikingly, ILCs also exhibit features of innate memory. NK cells display classical memory responses, while ILC1s and ILC2s can acquire trained immunity-like phenotypes characterized by enhanced and more rapid cytokine production upon secondary stimulation and expression of genes typically associated with classic T cell memory (7, 16, 20, 21). These memory-like properties confer adaptability to ILCs across diverse tissues and contribute to long-term immune modulation.

Despite their promise, studying ILCs presents substantial practical challenges due to their scarcity, tissue residency, phenotypic plasticity, and lack of definitive markers. They are scarce and primarily tissue-resident, making them difficult to isolate, study, and expand for clinical use. This likely explains why they remained largely undetected until the late 2000s. Their frequency in peripheral blood is much lower than that of T cells or NK cells, limiting feasible collection and analysis from routine blood draws (22). Collecting tissue-resident ILCs requires invasive biopsies from sites such as the gut mucosa, lung, or skin, invoking ethical and logistical constraints and reducing sample availability in human studies. Consequently, most clinical studies rely on circulating ILCs, which are scarce and may not accurately reflect local immune dynamics at sites of inflammation. Circulating ILCs often differ phenotypically and functionally from their tissue-resident counterparts, providing only a limited systemic snapshot of disease activity. In addition, ILC phenotyping demands precise lineage-negative gating, and even minimal contamination from T cells can skew results, complicating the reproducibility of functional assays. Moreover, as noted above, *in vitro* culture and exposure to cytokines can substantially alter their phenotype thereby masking their true behavior in tissues. Accordingly, much of the mechanistic understanding of ILCs derives from murine models; however, key differences between mouse and human ILC phenotypes, subset distribution, and cytokine responsiveness impede direct clinical translation (22). *Ex vivo* expansion methods, such as differentiation from umbilical cord blood (CB)-derived CD34^+^ progenitors, addresses some limitations by enabling generation of NK, ILC2, and ILC3 subsets from a renewable source (22). However, these platforms remain at an early stage and face challenges related to scalability, functional fidelity, and regulatory validation. Adoptive transfer of primary ILCs has shown promise in murine graft-versus-host disease (GVHD) models, yet limited cell yields, the enzyme sensitivity of ILCs, and phenotypic instability in immunodeficient hosts currently constrain therapeutic reproducibility (22). Moreover, the intrinsic plasticity of ILCs and their responsiveness to microenvironmental cues further complicate efforts to maintain stable and functionally defined cell products during expansion, storage, and clinical delivery. These challenges highlight the need for continued mechanistic insights into ILC biology and for the development of innovative strategies to overcome current technical and translational barriers.

### 1.2 ILC ontogeny: a multilayered model of development

ILC development is increasingly recognized as a multilayered developmental process, with distinct waves of progenitors and mature cell types arising from the yolk sac, bone marrow (BM), and thymus. This multi-origin model mirrors the developmental architecture of T cells and helps explain the diversity and tissue distribution of ILC subsets across the lifespan. Embryonically, the yolk sac contributes the earliest wave of ILC progenitors. Ni et al. demonstrated that human yolk sac–derived multipotent progenitors with innate lymphoid bias emerge even before definitive hematopoietic stem cells (HSCs), seeding embryonic tissues with tissue-resident ILC populations (23). These embryonic ILCs resemble tissue-resident γδ T cells and contribute to local immune niches independently of later BM hematopoiesis. Additionally, Sun et al. (2023) demonstrated *in vitro* that fetal hematopoietic progenitors and human pluripotent stem cells can give rise to early lymphoid populations, further supporting a layered model of ILC development spanning embryonic and fetal stages (24).

In adulthood, bone marrow (BM) has been proposed to be the primary and continuous source of ILCs. According to this model, hematopoietic stem and progenitor cells (HSPCs) in the BM give rise to distinct ILC precursor populations (ILCPs and NKPs) that differentiate under the control of key transcription factors (25). NFIL3 initiates ILC lineage commitment by inducing ID2, while downstream factors such as RORα and RORγt are crucial for subset specification (26). Abe et al. showed that RORα is indispensable for adult BM-derived type 1 ILC (ILC1/NK) development (27), while ILCPs remain responsive to local cues and can be mobilized during inflammation (28). While the dependency of these cells on these key transcription factors is clear, recent fate mapping data suggest that BM ILC subsets may, in fact, represent tissue resident ILC subsets derived from ILC precursors that seeded tissues during early neonatal development and therefore this dogmatic view may need revision (29, 30).

Recent thymic studies add an important new layer to this model. Miyazaki et al. showed that thymic Notch–E2A circuitry not only promotes αβ T cell fate but also actively suppresses helper ILC potential, particularly ILC2 and ILC3 lineages; when this repression is relieved, thymic progenitors can adopt ILC fates (31). Shin et al. provided direct evidence that normal thymic T cell precursors can give rise to ILC2s, with abortive γδ-TCR rearrangements indicating that some thymic ILC2s derive from T lineage precursors (32). These studies are in contrast to the earlier dogmatic view that all ILCs arise directly from bone marrow precursors that then colonize peripheral tissues directly. The newer studies also reinforce previous observations from Takei and colleagues showing frequent abortive γδ-TCR rearrangements in “NK cells” which, in retrospect, likely represent an admixture of NK cells and ILC1s (33). Based on these observations, Shin & McNagny further emphasized the thymus as a potential source of ILCs and have called for a re-evaluation of thymic contributions to the peripheral adult ILC compartments (34). Jan-Abu et al. expanded on this concept, highlighting parallel developmental pathways between T cells and ILCs within the thymus and proposing that thymic progenitors can contribute to ILC lineages under certain contexts (35). Complementing these findings, Ferreira et al. demonstrated that RORα serves as a critical checkpoint enabling ILC2 commitment from T cell precursors in the embryonic thymus (36). Pease et al. further demonstrated that a timed epigenetic switch, involving dynamic changes in chromatin accessibility, regulates the balance between T cell and ILC lineage output in the thymus; this mechanism ensures proper lineage proportions by progressively limiting ILC potential as thymopoiesis proceeds (37). In summary, the latest data suggest a thymic origin of many ILC subsets and that the same regulatory mechanisms that govern T cell subset specification from early thymic progenitors (ETPs) also are play in the commitment of ILCs.

ILC ontogeny, thus, reflects a multifocal and temporally layered progression: fetally derived progenitors establish early tissue-resident pools that expand and contract homeostatically in response to local cues; adult BM, as a source of HSC, could serve as an ultimate source of all hematopoietic precursors including CLPs and ILCs and thereby sustain lifelong ILC renewal; the thymus and ETPs serving as an additional, regulated source of ILCs. The relative contributions of these varies sources to tissue resident populations of ILCs in disease scenarios remains to be clarified. That point aside, these new insights into the developmental continuum parallels that of adaptive T cells, with NK cells and γδ T cells representing cytotoxic, tissue-resident branches, and helper ILCs (ILC1/2/3) resembling Th1/2/17 functional programs across tissues.

### 1.3 Therapeutic relevance of helper ILCs in immune-mediated diseases

ILCs are increasingly recognized as central regulators of immune responses across a broad spectrum of diseases, including cancer, autoimmune, and chronic inflammatory conditions. While clinically distinct, these diseases share a common underlying feature—immune imbalance—in which ILCs can act as either protectors or drivers of pathology. In autoimmune diseases, dysregulated ILC activity is increasingly recognized as a contributor to chronic inflammation and tissue damage; elevated ILC1 and ILC3 populations have been implicated in conditions such as systemic lupus erythematosus and rheumatoid arthritis (38, 39). Similarly, in inflammatory bowel disease, aberrant activation of gut-resident ILC3s exacerbates local inflammation and disrupts epithelial integrity (38–40). In cancer, ILC subsets may be co-opted by the tumor microenvironment to suppress antitumor immunity or promote tumor growth, with IL-17 cytokine family contributing to angiogenesis and tissue remodeling (41, 42).

A recurring theme across these diseases is the dysregulated production of cytokines—including IL-1β, IL-6, IL-17, and IL-22—by tissue-resident ILCs. While these mediators are essential for host defense and tissue repair, their overproduction drives chronic inflammation, fibrosis, and tissue remodeling. Compounding this dysregulation is the suggestion of remarkable ILC subset plasticity; influenced by signals such as IL-12 and TGF-β, ILCs can dynamically shift phenotype and function, further modulating disease trajectories (43–45).

Although NK cells, which are classified within the broader ILC family, have been extensively studied as targets for adoptive cell therapy, with significant progress in *ex vivo* expansion, differentiation from pluripotent stem cells, and CAR-NK engineering for cancer treatment, the therapeutic development of helper ILC subsets (ILC1, ILC2, ILC3, and LTi cells) remains comparatively underexplored. Several recent reviews highlight that NK cell-based therapies are now entering clinical practice with encouraging results, particularly for hematologic malignancies, and are being actively optimized to overcome barriers in treating solid tumors (46–49). While underscoring that NK cell therapy platforms are maturing rapidly, in contrast, the therapeutic potential of helper ILCs remains largely untapped, despite their unique immunomodulatory, regenerative, and tissue-resident properties that could complement or extend current cell-based approaches. Accordingly, this review is staged to focus on helper ILCs, aiming to bridge this gap and to advance our understanding of helper ILC’s potential as next-generation therapeutic agents in autoimmune diseases and cancer. The following sections will examine the underpinnings of helper ILCs in immune-mediated diseases and discuss how emerging strategies may unlock their potential as next-generation targets for precision cell-based interventions.

While numerous reviews have covered individual aspects of ILC biology, a comprehensive synthesis integrating their multifaceted roles in both autoimmune diseases and cancer, alongside a critical assessment of emerging engineering and omics platforms for therapeutic translation, remains lacking. With this review, we aim to bridge this critical gap by providing a comprehensive analysis of helper ILCs, highlighting their immunoregulatory functions, phenotypic plasticity, and interactions within tissue microenvironments. In it, we also evaluate how advances in pluripotent stem cell–derived models, synthetic biology, metabolic profiling, and single-cell multi-omic technologies are reshaping the translational potential of ILCs. By connecting fundamental biology with applied strategies such as CAR and CSR engineering, immune checkpoint modulation, and regenerative interventions, this review offers a framework for positioning helper ILCs as potential targets in next-generation precision immunotherapy.

### 1.4 Literature search strategy and selection criteria

This review was conducted in accordance with the PRISMA Extension for Scoping Reviews (PRISMA-ScR) guidelines to ensure transparency, reproducibility, and scientific rigor in synthesizing the roles and therapeutic potential of helper ILCs in autoimmune diseases and cancer.

We systematically searched PubMed, Scopus, and Web of Science for peer-reviewed, English-language literature published between January 2000 and June 2025. This time frame was selected to capture both recent advances in innate lymphoid cell biology and foundational discoveries that have shaped current understanding, reflecting a period of sustained conceptual and translational growth in the field.

The search strategy combined Medical Subject Headings (MeSH) and free-text terms. The base search string used across all databases was: (“innate lymphoid cells” OR “ILCs”) AND (“autoimmune diseases” OR “cancer” OR “tumor” OR “immunotherapy” OR “CAR-ILC” OR “PSC-derived ILCs”). Additional targeted searches were performed using disease-specific and mechanistic combinations such as “ILC3 AND colorectal cancer AND checkpoint blockade” or “ILC2 AND systemic lupus erythematosus AND IL-33” to extract deeper mechanistic insights and explore translational applications. These iterative queries were guided by findings from the initially retained studies to ensure conceptual depth and disease relevance.

Studies were included if they focused on helper ILC subsets (ILC1s, ILC2s, ILC3s, or LTi cells), presented original data or comprehensive mechanistic reviews, addressed roles in autoimmune diseases and/or cancer, and provided insight into therapeutic applications or platforms, including CAR/CSR engineering, immune modulation, or CB/PSC-derived ILCs. Studies were excluded if they were non-English, unpublished, preprints, editorials, conference abstracts, or lacked peer review. Articles focusing exclusively on NK cells were excluded unless they offered comparative insight relevant to helper ILCs, and papers not centered on ILC biology or lacking translational value were also removed.

Screening was performed independently by the first author, with additional publications suggested by co-authors. Titles and abstracts were reviewed for initial relevance, followed by full-text evaluation to confirm eligibility. Discrepancies and uncertainties were resolved through full-text comparison and cross-verification using Mendeley reference management software. Relevance and quality were assessed based on journal impact, mechanistic depth, and translational significance.

Of the more than 230 references cited in this review, a curated subset of 52 studies formed the primary evidence base used for the mechanistic synthesis and therapeutic discussions presented in sections 2 through 4. These studies were selected for their high-quality mechanistic detail, clinical or translational relevance, and balanced coverage across autoimmune and cancer-related contexts.

Although formal risk-of-bias assessment tools were not applied due to the heterogeneous nature of the included studies, the review prioritized peer-reviewed, methodologically rigorous research. Preprints and grey literature were excluded to preserve data reliability and interpretative clarity.

Lastly, we acknowledge that, despite our best efforts, in any compendium of a large body of scientific literature there is an inherent bias toward topics of specific scientific interest to the authors and that this may have inadvertently biased the authors toward those impactful articles that have shaped our own thinking and perspectives.

## 2 ILCs in autoimmune and inflammatory diseases

Autoimmune and inflammatory diseases encompass a broad spectrum of disorders in which the immune system aberrantly targets self-tissues or drives chronic inflammation in response to innocuous antigens. Traditionally, conditions such as type 1 diabetes (T1D), multiple sclerosis (MS), rheumatoid arthritis (RA), and systemic lupus erythematosus (SLE) have been classified as classic autoimmune diseases, mediated primarily by autoreactive T and B lymphocytes. In contrast, diseases such as inflammatory bowel disease (IBD), systemic sclerosis (SSc), and antineutrophil cytoplasmic antibody (ANCA)-associated vasculitis (AAV) exhibit mixed pathogenesis, with substantial contributions from innate immune dysregulation and autoinflammatory mechanisms (50, 51). Across this disease spectrum, ILCs have emerged as key modulators of immune responses, influencing disease initiation, progression, and resolution. Dysregulation of ILC numbers, phenotypes, and functions has been reported in many autoimmune and inflammatory conditions (2, 5, 6). For example, expansion of ILC1s correlates with heightened inflammatory activity in MS, SLE, and AAV (52, 53); aberrant ILC2 responses contribute to fibrosis in SSc and impaired tissue repair in SLE (54, 55); and plasticity or imbalance of ILC3 subsets disrupts gut barrier integrity and modulates systemic immune responses in IBD, RA, and T1D (56–58).

While the role of ILCs in autoimmune and chronic inflammatory diseases is increasingly well defined, their involvement in primary autoinflammatory diseases, such as familial Mediterranean fever and other inflammasome-driven syndromes, remains largely unexplored (50, 51). Given that autoinflammatory diseases are mediated predominantly by innate immune dysregulation, the potential contributions of ILCs represent an important but under-investigated area of research. Accordingly, this review focuses specifically on the emerging roles of ILCs in autoimmune diseases and cancer, where current insights into therapeutic opportunities are most advanced.

With this understanding, the therapeutic potential of targeting ILCs is increasingly recognized. Modulating ILC function through cytokine blockade, regulation of plasticity, inhibition of migration, or manipulation of metabolic and microbial pathways could complement existing therapies and offer new treatment avenues (2, 59). ILCs may also serve as valuable biomarkers for disease severity, progression, and response to therapy. However, current reliance on peripheral blood assessments limits our ability to fully characterize the functional roles of ILCs in autoimmune pathogenesis, particularly within affected tissues (2).

Despite these challenges, ongoing advances in ILC biology across autoimmune and inflammatory diseases offer exciting opportunities for precision immunotherapy and the following sections examine the roles of ILCs in T1D, MS, SLE, AAV, RA, SSc, and IBD, highlighting both disease-specific mechanisms and broader immunological themes (**Table 1**).

**Table 1:** Functional roles of ILC subsets in autoimmune and inflammatory diseases.

Innate lymphoid cells (ILCs) in autoimmune diseases			
Disease name	ILC1	ILC2	ILC3
Type 1 Diabetes (T1D)	Enhanced frequencies correlated with mucosal dysfunction and systemic immune dysregulation (60, 61)	IL-13 and CSF-2 promote pancreatic homeostasis (59, 62)	IL-22 indirectly enhances epithelial barrier integrity and protects β-cells from oxidative and ER stress, IL-2 supports Treg function (63)
Multiple Sclerosis (MS)	IL-10 producing CCR6^+^ subset significantly reduced in patients (64, 65)	Contributes to tissue protection and repair, significantly reduced in patients (65, 66)	Presents antigen to CD4 T cells, amplifying T-cell driven neuroinflammation (67, 68)
Systemic Lupus Erythematosus (SLE)	Frequency increased in patients; produce IFN-γ, amplifies systemic inflammation and organ damage (38, 69–71)	Maintain barrier integrity via IL-5 and IL-13; consistently reduced in patients; can convert into pro-inflammatory ILC1 phenotype via IL-12 and IL-1β; IL-33-induced activation promotes eosinophilic infiltration and fibrosis (liver), contributes to pulmonary and dermal fibrosis (38, 39, 72–76)	NCR^+^ ILC3: produce IL-22 to maintain epithelial integrity, provide protection in gut and liver (77–80) NCR^-^ ILC3: secrete IL-17A, GM-CSF and CCL4, exacerbate inflammation, promote M1 macrophage polarization, contribute to tissue injury and fibrosis in gut and liver (54, 77, 81, 82)
Antineutrophil Cytoplasmic Antibody (ANCA)-Associated Vasculitis (AAV)	Exacerbate neutrophil-driven vascular inflammation (83, 84)	Promote tissue repair through IL-5 and IL-13 (83, 85)	Promote mucosal homeostasis through IL-22 (83, 85)
Rheumatoid Arthritis (RA)	Increased in lymph nodes and synovial fluid; promote inflammation through JAK/STAT signaling and IFN-γ production (55, 86)	Higher levels observed in stable patients; produce IL-9 and IL-13; suppress synovitis, support macrophage modulation, promote vascular and tissue repair (87–90)	ILC3 produce IL-17 and IL-22, promote Th17 differentiation, drive local inflammation and correlate with clinical disease activity (91, 92) LTi cells reduced in RA lymph nodes; frequency correlates with VCAM-1 expression on endothelial cells; contribute to lymphoid tissue maintenance and stromal cell–ILC interactions (86)
Systemic Sclerosis (SSc)	Increased in circulation; sustain chronic inflammation via IL-6 and IFN-α pathways (38, 93, 93)	Increased in skin and blood; KLRG1 ^low^ subset gets activated through TGF-β and promotes fibrosis (38, 94–97)	NKp44^+^ subset increases and contributes to inflammation and fibroblast activation; plasticity with ILC2 under IL-23 and IL-1 family cytokines (38, 93)
Inflammatory Bowel Disease (IBD)	Expanded in inflamed mucosa; promote barrier dysfunction, fibrosis, and inflammation; arise from ILC3-to-ILC1 plasticity (55, 56, 98–102)	Promote epithelial repair and barrier integrity; can adopt pro-fibrotic and pro-inflammatory phenotypes in response to IL-33 and microbiota signals (103–105)	Dysregulated NCR^-^ ILC3s promote IL-17A–driven inflammation and barrier damage; plasticity toward ILC1 fuels chronic inflammation (32–42, 106–109)

Protective and pathogenic functions of helper-type innate lymphoid cells (ILC1s, ILC2s, and ILC3s) across autoimmune and inflammatory conditions including T1D, type 1 diabetes; MS, multiple sclerosis; SLE, systemic lupus erythematosus; AAV, antineutrophil cytoplasmic antibody-associated vasculitis; RA, rheumatoid arthritis; SSc, systemic sclerosis; IBD, inflammatory bowel disease.

### 2.1 ILCs in type 1 diabetes

Type 1 diabetes (T1D) is a chronic autoimmune disease characterized by the destruction of pancreatic β-cells, leading to insulin deficiency and lifelong dependence on exogenous insulin (110). ILCs represent a rapidly expanding area of investigation in the context of T1D. These innate immune populations are present in the pancreas both at steady state and during disease progression in murine models (111–113). Human studies have indicated altered NK cell phenotypes in T1D patients, though the precise role of NK cells in T1D remains unresolved (62, 114). Most studies to date have evaluated NK cells in bulk, without discriminating between functionally distinct subsets such as CD56^high^, CD56^low^, or regulatory NK-like cells (115), complicating interpretation. While some data suggest cytotoxic NK cells may contribute to β-cell destruction, other studies have implicated NK-derived regulatory pathways that could promote tolerance (116).

In contrast to the ambiguous role of NK cells, accumulating evidence supports the view that helper-like ILCs, particularly ILC2s and ILC3s, contribute to pancreatic homeostasis and exert protective effects in the setting of autoimmunity (117). IL-33 produced by pancreatic mesenchymal cells activates resident ILC2s, promoting their secretion of IL-13 and colony-stimulating factor-2 (CSF-2, aka GM-CSF). These factors stimulate retinoic acid production by dendritic cells (DCs) and macrophages, which in turn enhances insulin secretion from β-cells (111). Mechanistically, retinoic acid serves as a ligand for nuclear retinoic acid receptors (RARs) in β-cells, where it promotes insulin gene transcription and β-cell function (117). The protective role of ILC2s extends beyond the pancreas: in models of type 2 diabetes, ILC2-derived IL-5 maintains eosinophils and alternatively activated macrophages in adipose tissue, reducing systemic insulin resistance (63). These observations suggest that ILC2-mediated regulation of tissue-resident myeloid cells may represent a conserved mechanism for maintaining metabolic homeostasis across tissues.

ILC3s also appear to play a key role in regulating gut–pancreas immune crosstalk and preventing autoimmune diabetes. Commensal-driven stimulation of ILC3s induces IL-22 production, which enhances epithelial barrier integrity and drives β-defensin 14 (mBD14) expression in pancreatic endocrine cells. mBD14 promotes proliferation and recruitment of regulatory B cells and tolerogenic macrophages, fostering a regulatory milieu that limits β-cell autoimmunity in non-obese diabetic (NOD) mice (112). IL-22 also protects β-cells from oxidative and ER stress (60), though its therapeutic utility remains debated (61). Beyond pancreatic effects, gut-resident ILC3s integrate microbial and dietary signals within gut-associated lymphoid tissue (GALT), producing IL-22 to maintain epithelial integrity and IL-2 to support regulatory T cell (Treg) function (118). Reductions in ILC3 and Treg frequencies, alongside impaired gut barrier function, are linked to T1D pathogenesis, highlighting the importance of ILC3–Treg circuits in controlling systemic autoimmunity (118). Targeting free fatty acid receptor 2 (FFAR2), highly expressed on intestinal ILC3s, with synthetic agonists or dietary interventions, offers a promising strategy to boost ILC3 protective functions and attenuate β-cell autoimmunity (118). Consistent with this, T1D patients exhibit elevated ILC1 and diminished ILC3 frequencies in the duodenum (67), and this likely contributes to mucosal dysfunction and systemic immune dysregulation. Furthermore, ILC3 plasticity whereby ILC3s adopt pro-inflammatory ILC1-like phenotypes may exacerbate autoimmune inflammation (119). ILC–adaptive immune crosstalk remains a critical axis influencing T cell polarization, regulatory networks, and antigen-presenting cell function in autoimmunity (40).

Current evidence positions ILCs as key modulators of pancreatic immune homeostasis with both pathogenic and protective potential in T1D. While NK cell functions remain incompletely defined, ILC1/ILC3 balance and ILC2/3-driven regulatory circuits appear to preserve β-cell function and support pancreatic immune tolerance and may be utilized therapeutically to prevent or delay disease progression. Future work should focus on characterizing human pancreatic ILC networks across T1D stages, defining the interplay between ILCs and adaptive immune populations, and developing ILC-based interventions for both endogenous β-cell preservation and improved islet graft outcomes.

### 2.2 ILCs in multiple sclerosis

Multiple sclerosis (MS) is an autoimmune neurodegenerative disease characterized by central nervous system (CNS) inflammation and demyelination. Although traditionally regarded as a T-cell–mediated disease, ILCs are increasingly recognized as key modulators of pathological immune responses in MS (68). ILCs are typically tissue-resident cells; however, subsets can infiltrate the CNS during neuroinflammation, where they actively shape disease progression. In particular, CNS-infiltrating ILC3s function as antigen-presenting cells (APCs), re-stimulating myelin-specific CD4^+^ T cells through MHCII-TCR interactions, thereby amplifying T-cell–driven neuroinflammation. Notably, these ILC3s upregulate classical MHC II molecules (e.g., H2-Ab1/Aa in mice, HLA-DR in humans) and co-stimulatory markers such as CD80 and CD86 during CNS inflammation, equipping them with key features of professional APCs (66). This APC activity is required for disease progression in experimental autoimmune encephalomyelitis (EAE), the murine model of MS, and correlates with increased ILC3 abundance in MS patients. Furthermore, meningeal accumulation of ILCs is an early event during lesion formation in both MS and EAE (120), highlighting their capacity to initiate and perpetuate CNS autoimmunity.

Importantly, ILC function in MS is highly context dependent. While ILC3s and cytotoxic NK cells can exacerbate neuroinflammation (64), other subsets including ILC2 and CD56^high^ NK cells, contribute to tissue protection and repair (121). Physiological states such as pregnancy, which is associated with reduced MS relapse rates can promote beneficial ILC shifts. *In vitro* exposure of MS patient–derived ILCs to estriol and commensal bacterial strains (*Escherichia coli* K12 and *Lactobacillus plantarum* 8R-A3) induced regulatory ILC transitions, enhancing anti-inflammatory phenotypes (65). Similarly, retinoic acid, an immunoregulatory metabolite of vitamin A, promotes expansion of IL-10-producing ILC1 while reducing CNS-migratory CCR6^+^ ILC2 and ILC3 subsets, further supporting a role for metabolite-driven modulation of ILC plasticity or other subset expansion (122). ILC migration into the CNS is also shaped by an evolving understanding of CNS immune interfaces. The glymphatic system and meningeal lymphatic vessels provide pathways for CNS antigen drainage and immune cell trafficking to peripheral lymphoid tissues (69, 123). In MS patients, circulating CCR6^+^ ILC1 and ILC2 subsets are significantly reduced, consistent with active migration into the CNS (69). Moreover, ILCs engage in dynamic interactions with CNS stromal and barrier cells, influencing immune surveillance and local neuroimmune homeostasis (70, 71).

Given their plasticity and/or ability to rapidly expand and contract in response to environmental cues, ILCs act as both drivers and modulators of neuroinflammation and represent an attractive therapeutic target in MS. Selective blockade of pathogenic CNS-infiltrating ILC3s by blocking their migration or antigen-presenting capacity, enhancement of protective ILC2 and CD56^high^ NK cell responses, and modulation of ILC migration via CCR6 or glymphatic pathways, could offer promising complement to existing T- and B-cell–focused therapies (68). In addition, retinoic acid, estriol, microbiota- and metabolomic-based interventions can offer opportunities to reshape peripheral ILC composition/plasticity/expansion, potentially restoring immune homeostasis and limiting disease progression.

### 2.3 ILCs in systemic lupus erythematosus

In systemic lupus erythematosus (SLE), a complex multi-organ autoimmune disease characterized by persistent inflammation and autoantibody production, dysregulation of ILC subsets is increasingly recognized as a critical contributor to pathogenesis. Multiple studies have shown significant alterations in circulating ILCs (cILCs), particularly during active disease phases. Chu et al. and Jiang et al. observed increased frequencies of ILC1s in the peripheral blood of SLE patients, with positive correlations to disease activity and renal involvement. These pro-inflammatory ILC1s, primarily producing IFN-γ, may amplify systemic inflammation and thereby contribute to organ damage (72, 124, 125). In contrast, ILC2s which are more frequently associated with tissue repair via production of IL-5 and IL-13, are consistently reduced in SLE, suggesting impaired mechanisms for controlling inflammation and promoting mucosal and epithelial healing (40, 41). ILC3s are variably altered, with increases often correlating with heightened inflammation and tissue infiltration (126).

In visceral organs such as the kidneys and liver, ILCs contribute significantly to tissue injury, loss of function and fibrosis. For example, in lupus nephritis, a common and severe manifestation of SLE, upregulation of CD56^high^ and CD56^low^ NK cells and ILC1s leads to enhanced secretion of IFN-γ and TNF, driving glomerular inflammation (40, 125). ILC2s, which normally dominate the renal ILC pool and secrete anti-inflammatory cytokines like IL-5 and IL-13, are suppressed in lupus nephritis. Administration of IL-33 can restore ILC2 function and ameliorate nephritis, but prolonged stimulation may trigger fibrosis via amphiregulin (AREG) and excessive IL-13 production (73, 77). In the liver, autoantibody-mediated damage induces IL-33 release, activating ILC2s to secrete IL-15 and IL-13, promoting eosinophil infiltration and hepatic fibrosis (73, 74). While NCR^+^ ILC3s provide protection through IL-22 production, NCR^-^ ILC3s exacerbate liver injury via IL-17A secretion, and IL-17 blockade shows therapeutic promise (75).

Barrier tissues such as the lungs and skin also exhibit ILC-driven pathology in SLE. In pulmonary tissues, ILC3s predominate and support antimicrobial defense via IL-17 and IL-22. However, chronic inflammation converts ILC2s into ILC1s through IL-12 and IL-1β signaling, increasing IFN-γ and worsening pulmonary fibrosis (76, 81, 82). Also, IL-25-induced activation of ILC2s augments IL-13 secretion, further promoting fibrotic tissue remodeling (78). In the skin, ILC2s dominate the upper dermis and can be activated by epithelial-derived IL-25 and IL-33. These cells secrete IL-5 and IL-13, contributing to epidermal barrier disruption and chronic Th2-type inflammation (40, 126). They also impair keratinocyte formation and tight junction integrity, exacerbating cutaneous lupus. While IL-33-induced ILC2s support wound healing, their sustained activation drives dermatitis via a pathological chronic repair response. Therapeutics such as dupilumab, which suppress ILC2 and Th2 activity by blocking IL-4Rα and inhibiting IL-4 and IL-13 signaling, have demonstrated clinical benefit in reducing skin inflammation. However, dampening ILC2-mediated responses may also impair tissue repair functions, increase susceptibility to infections, and disrupt immune homeostasis (41, 78).

In the gastrointestinal tract, ILC1s and ILC3s play central roles in regulating inflammation and commensal microbiota balance. Inflammatory circuits involving ILC1- and ILC3-derived cytokines activate myeloid cells and sustain local immune responses. ILC1-derived IFN-γ promotes pro-inflammatory macrophage polarization, while ILC3-derived GM-CSF drives recruitment, activation, and survival of monocytes and macrophages, contributing to persistent inflammation (56, 79, 80). In addition, ILC3s, together with RORγt^+^ DCs, present antigens to T cells via MHC class II (MHCII) and promote epithelial barrier integrity through IL-22 production (83, 84, 127). Dysbiosis and epithelial damage in SLE trigger aberrant ILC3 activation, leading to excessive IL-17, IL-22, and CCL4 production that worsens gut inflammation (41). ILC2s help maintain barrier integrity by regulating goblet and tuft cells, and their reduction may exacerbate microbial translocation and systemic autoimmunity.

Collectively, the tissue-specific roles of ILCs in SLE emphasize their paradoxical nature where they are protective under homeostatic conditions but pathogenic when dysregulated. Targeting key signaling axes, such as IL-33/ST2, IL-12/IL-1β, and IL-17/IL-22 offers promising therapeutic avenues. By restoring ILC subset balance and function, it may be possible to mitigate organ-specific damage and improve outcomes in SLE patients without systemic immunosuppression (40, 41, 82, 124, 126).

### 2.4 ILCs in antineutrophil cytoplasmic antibody-associated vasculitis

Antineutrophil cytoplasmic antibody (ANCA)-associated vasculitis (AAV) encompasses a group of autoimmune disorders—including granulomatosis with polyangiitis (GPA), microscopic polyangiitis (MPA), and eosinophilic granulomatosis with polyangiitis (EGPA)—characterized by inflammation and necrosis of small to medium-sized blood vessels. Its pathogenesis is primarily attributed to autoantibody-mediated neutrophil activation (85). Emerging evidence suggests that ILCs also modulate AAV. Several studies report dynamic changes in circulating ILC subsets during active disease phases: increased ILC1 frequencies and decreased ILC2 and ILC3 in patients with active GPA/MPA versus healthy controls. These alterations tend to normalize in remission, linking ILC subtype distribution to disease activity (86, 91).

Specifically, circulating ILC1 expansion may fuel inflammation in active AAV, while reductions in ILC2/ILC3s—cells key to tissue repair and mucosal homeostasis via cytokines like IL-5, IL-13, and IL-22—might impair tissue recovery and exacerbate vascular damage (86, 92). However, most of these data derive from assessment of peripheral blood ILCs, and the roles of tissue-resident ILCs in affected organs remain largely unexplored. Recent data by Bennstein & Uhrberg et al. supports these patterns: active AAV is marked by ILC1 upregulation and ILC2/ILC3 downregulation, which return toward baseline during remission, reinforcing the hypothesis of ILC subset shifts serve as important biomarkers of disease activity. Despite this, the mechanistic pathways through which ILCs contribute to vasculitic damage remain unclear, especially regarding how tissue-resident ILCs interact with endothelial cells and neutrophil-driven inflammation (91).

In summary, current data indicate a correlation between circulating ILC subset imbalances and AAV activity. Confirmation of ILC causality and therapeutic potential of targeting them requires deeper investigation—particularly into organ-resident ILC subsets and dynamics and their communication with vascular microenvironments.

### 2.5 ILCs in rheumatoid arthritis

ILCs are increasingly recognized as critical players in rheumatoid arthritis (RA), an autoimmune disease characterized by persistent synovial inflammation and progressive joint destruction. Multiple studies have reported shifts in ILC subset distributions in RA patients, suggesting their possible roles in disease modulation. Rodriguez-Carrio et al. observed significant alterations in lymph node–resident ILC populations, with reductions in lymphoid tissue inducer (LTi) cells and increases in ILC1s and ILC3s compared to healthy controls and at-risk individuals (87). LTi frequency positively correlated with VCAM1 expression on lymph node endothelial cells, underscoring stromal–ILC interactions. In inflamed synovial fluid, Takaki-Kuwahara et al. found enrichment of CCR6^+^ ILC3s that correlated with clinical disease activity and CCL20 levels, likely through IL-17/IL-22–driven local inflammation (88). Complementing this, Liu et al. demonstrated that ILC3s promote Th17 differentiation in RA through direct interactions with T cells, reinforcing ILC3’s role in perpetuating synovial inflammation (89).

In contrast, ILC2s appear to have protective effects. Yang et al. reported that RA patients with stable disease had higher peripheral ILC2 levels and lower ILC1 proportions, with ILC2 frequency inversely correlating with disease activity. The anti-inflammatory functions of ILC2s are thought to involve IL-9 and IL-13, which suppress synovitis via macrophage modulation in RA models (55, 90, 128, 129). Mechanistically, ILC2–vascular/stromal crosstalk—via chemokine receptor pathways—has been shown to promote vascular repair and tissue homeostasis in chronic inflammation (89), suggesting similar protective roles in RA synovia. However, the line between pathogenic and protective ILC roles is blurred by substantial plasticity shaped by the local microenvironment. This plasticity is further illustrated in IBD, where modulation of cytokine networks (e.g., anti-IL-12/23 ustekinumab) partially restores ILC subset balance and shifts pro-inflammatory ILC1/ILC3 populations toward a more homeostatic profile (130). Supporting this, Lo Pizzo et al. also demonstrated that JAK/STAT inhibition with tofacitinib selectively modulates ILC1-driven IFN-γ production in RA patients, highlighting therapeutic potential for targeting specific ILC functions (131). However, because JAK inhibitors broadly suppress multiple cytokine pathways (e.g., IL-15, IL-7, IL-21, type I/II IFNs), they may also impair mucosal barrier integrity and antiviral defenses, increasing susceptibility to infections such as herpes zoster, bacterial pneumonia, and reactivation of latent infections (132–135).

Overall, these findings highlight ILC subsets—particularly ILC3 and ILC2—as both biomarkers and potential therapeutic targets in RA. They underscore the need for functional investigations into ILC–stromal and ILC–vascular interactions, plasticity mechanisms, and subset-specific modulation strategies to influence disease outcomes.

### 2.6 ILCs in systemic sclerosis

Systemic sclerosis (SSc) is an autoimmune connective tissue disease characterized by vasculopathy, immune dysregulation, and progressive fibrosis affecting the skin, lungs, and vasculature (94–96, 136, 137). While the involvement of adaptive immune cells in promoting fibrosis is well established, emerging studies implicate ILCs as important modulators of SSc pathogenesis. In particular, ILC2s appear to play a key profibrotic role. In 2016, Wohlfahrt et al. demonstrated that ILC2 numbers are significantly elevated in both skin and peripheral blood of SSc patients compared to healthy controls (97). Notably, ILC2 frequencies are also positively correlated with the modified Rodnan Skin Score (mRSS), a clinical score of dermal disease severity, and the extent of interstitial lung disease, with the highest levels observed in patients with diffuse cutaneous SSc and severe pulmonary fibrosis. ILC2s were identified using both ICOS^+^ST2^+^CD3^-^CD11b^-^ and ST2^+^IL-17RB^+^KLRG1^+^ marker profiles, indicative of a type 2 inflammatory state. More recent studies have further identified increased expression of ILC2 activation markers such as IL-17RB and thymic stromal lymphopoietin receptor (TSLPR) on skin-homing ILC2s, suggesting an ongoing profibrotic activation state (40, 93). Mechanistic insights by Laurent et al. revealed that exposure to TGF-β induces a phenotypic switch in skin-resident ILC2s from KLRG1^high^ to KLRG1^low^ subsets. KLRG1^low^ ILC2s exhibited lower IL-10 production and a loss of their physiological capacity to suppress collagen synthesis by dermal fibroblasts. Instead, they promoted myofibroblast differentiation through secretion of profibrotic mediators such as IL-13 and amphiregulin, directly enhancing skin fibrosis by driving dermal fibroblast proliferation, αSMA induction, and collagen deposition (93, 138, 139). Combining IL-10 with pirfenidone restored KLRG1 expression and ameliorated fibrosis in an SSc mouse model (93). Longitudinal patient studies have since confirmed that elevated circulating ILC2s persist in patients with progressive SSc and correlate with adverse pulmonary and skin outcomes (140).

In parallel, group 1 ILCs (ILC1s) and ILC3s have also been implicated in SSc. Roan et al. reported increased frequencies of circulating ILC1s and NKp44^+^ ILC3s in SSc patients. ILC1s displayed reduced IL-6Rα expression, suggesting their role in sustaining chronic inflammation through ongoing IL-6 and interferon-alpha signaling (98). Additional biomarker studies have confirmed the presence of elevated circulating ILC1s in SSc without consistent changes in circulating ILC2s, highlighting dynamic regulation of ILC subsets (40, 99). These findings support a dual-pathogenic model wherein TGF-β-driven conversion of ILC2s into KLRG1^low^, IL-10-deficient effectors directly promotes fibrosis, while activated ILC1s sustain inflammatory circuits that perpetuate endothelial dysfunction and fibroblast activation. Moreover, ILC plasticity whereby ILC2s can acquire ILC1 or ILC3-like phenotypes under IL-12, IL-18, or IL-23 exposure could suggest dynamic reshaping of the ILC landscape during disease progression (40). While these data suggest ILCs as mechanistic drivers and potential biomarkers in SSc, key questions remain regarding their precise temporal roles, the molecular circuits governing their activation, and the potential for targeted modulation of ILC subsets to mitigate fibrosis. Future work integrating longitudinal patient cohorts with *in vitro* mechanistic studies and *in vivo* interventional models will be essential to realize the therapeutic potential of targeting ILCs in SSc.

### 2.7 ILCs in inflammatory bowel disease

Inflammatory bowel disease (IBD), encompassing Crohn’s disease (CD) and ulcerative colitis (UC), is a chronic inflammatory condition of the gastrointestinal tract characterized by disrupted mucosal homeostasis, altered immune responses, and microbial dysbiosis. Although often discussed together, CD and UC exhibit distinct immunopathologies: CD is typically associated with Th1/Th17-driven inflammation affecting the entire gut wall, while UC more often reflects Th2/ILC2-skewed responses limited to the colonic mucosa (100, 101). These differences likely shape ILC subset dynamics and plasticity across disease subtypes, contributing to the variability and sometimes contradictory findings in the ILC literature. ILCs are pivotal in maintaining intestinal tissue homeostasis, but their dysregulation contributes to IBD pathology (102). ILC1s, which support antiviral defense and homeostasis through IFN-γ and cytotoxic mediator production, expand aberrantly in IBD. Both intraepithelial and lamina propria ILC1s accumulate in inflamed mucosa, often at the expense of protective NKp44^+^ ILC3s, driven in part by IL-12–induced ILC3-to-ILC1 plasticity (57, 58). These activated ILC1s secrete IFN-γ, TGF-β1, granulysin, and MMP9, promoting epithelial barrier dysfunction, extracellular matrix remodeling, and fibroblast activation, thereby exacerbating fibrosis (103, 104). Moreover, ILC1-epithelial crosstalk sustains crypt hyperplasia and inflammation (105), while epigenetic regulation via TET enzymes modulates ILC1 proliferation (141). Depletion of pathogenic ILC1s alleviates colitis in experimental models (142), highlighting their pathologic relevance.

ILC2s contribute to both tissue repair and pathology in IBD through context-dependent mechanisms. Under homeostatic conditions, they promote epithelial regeneration via amphiregulin (AREG) and support barrier integrity through IL-5 and IL-13 (143). However, ILC2s are expanded in inflamed IBD mucosa, driven by elevated IL-33 and IL-25, and can adopt pro-fibrotic phenotypes. IL-13–producing ILC2s activate fibroblasts and promote extracellular matrix deposition (144), while diet-microbiota interactions, such as inulin-induced bile acid alterations, enhance IL-33 signaling and skew ILC2s toward pro-inflammatory IL-5 production, exacerbating disease (145). Vitamin B1 deficiency impairs IL-25-driven protective responses (146), and CCR2^+^ lung-derived ILC2s can migrate to the gut and contribute to inflammation (147). Furthermore, disrupted interactions with adaptive immune cells and altered aryl hydrocarbon receptor (AHR) signaling shift ILC2s toward pathogenic phenotypes (106, 107). AHR is a ligand-activated transcription factor that integrates environmental and microbial signals to regulate immune cell function (108, 109, 148). Notably, adoptive ILC2 transfer can mitigate colitis by enhancing epithelial repair (143), highlighting the dual potential of this subset in IBD.

ILC3s are critical for maintaining intestinal barrier integrity, immune tolerance, and microbiota regulation through IL-22, IL-17, GM-CSF, and MHCII-mediated T cell modulation (83, 149). In IBD, these functions become dysregulated. NCR^-^ ILC3s contribute to colitis via IL-23-driven IL-17A secretion, which recruits neutrophils and exacerbates epithelial barrier damage (42, 150, 151). IL-22, though protective by stabilizing the epithelial integrity, promoting mucus and antimicrobial peptide production (152), can also induce neutrophil-attracting chemokines (e.g., CXCL1, CXCL5) and drive pathology when overproduced (153, 154). ILC3 plasticity also fuels inflammation, with IL-12 and IL-2 from CD14^+^ DCs driving conversion of NKp44^+^ ILC3s into IFN-γ–producing ILC1s, depleting barrier-protective subsets (57, 58). Conversely, IL-23, IL-1β, and retinoic acid can restore ILC3 identity (58). AHR signaling further modulates ILC3 homeostasis, with its loss impairing NKp46^+^ ILC3 function and predisposing to colitis (155). Crosstalk with antigen-presenting cells via TL1A, IL-23, and IL-1β further fine-tunes ILC3 responses (153). Overall, ILC3s act as double-edged swords in IBD, providing essential barrier support yet contributing to chronic inflammation through context-dependent plasticity and cytokine production.

Current biologics largely modulate ILC activity indirectly. Anti-TNF therapy suppresses ILC1-derived IFN-γ, while vedolizumab, a humanized monoclonal antibody targeting α4β7, fosters a protective shift toward NCR^+^ ILC3s by targeting the α4β7–MAdCAM-1 axis, which regulates ILC migration (57). IL-23 blockade impacts ILC3 cytokine production, though paradoxical expansion of NCR^+^ ILC3s has been observed (156). Recent work identified neuropilin-1 (NRP1) as a key regulator of IL-17–producing ILC3s; its elevated expression in IBD mucosa promotes IL-17A production via NF-κB signaling (157). NRP1 blockade ameliorates colitis in preclinical models by limiting IL-17A production and altering microbiota composition (157). RORγt inhibitors, including bile acid metabolites such as 3-oxoLCA and isoLCA, and synthetic molecules like GSK805, offer another promising approach by preserving protective ILC3 subsets while reducing pathogenic Th17 responses (150, 158, 159). Additionally, TNF–TNFR and IL-17–IL-17R interactions are implicated in ILC effector functions and represent further potential targets (160). The TL1A–DR3 axis also modulates ILC2 and ILC3 activation and plasticity (161). Importantly, recent insights into ILC differentiation dynamics reveal that CD45RA^+^CD62L^+^ and CD62L^-^ naïve-like ILCs act as local precursors for tissue-resident ILC subsets, including IL-22–producing ILC3s that accumulate in inflamed IBD mucosa (162). These findings suggest that manipulating ILC precursor differentiation could represent a novel therapeutic avenue to restore mucosal homeostasis. Emerging strategies also aim to fine-tune ILC responses through cytokines such as IL-33 and IL-25 to enhance anti-inflammatory ILC2 activity (101), or through cytokine-driven modulation of ILC1/ILC3 plasticity to rebalance mucosal immunity (161). Other ILC modulators, including retinoic acid and lipoxin A4, show promise in promoting protective responses (160), while dietary and microbial signals profoundly influence ILC composition and function (101), offering opportunities for nutritional or microbiome-based interventions (156). However, major challenges remain as ILC heterogeneity and plasticity complicate therapeutic targeting. Additionally, some murine models fail to fully recapitulate human ILC dynamics (160). Finally, the roles of ILCs in fibrosis, fistula formation, and IBD-associated malignancies are still poorly understood (57, 162). Future therapeutic strategies should include development of tools for selective modulation of ILC subsets, longitudinal mapping of ILC behavior across disease stages, and integrative studies exploring ILC interactions with the microbiota, nervous system, and stromal environments. Critically, careful distinction between Crohn’s disease and ulcerative colitis, along with precise characterization of ILC subset dynamics within each disease context, will be essential for understanding how ILCs drive pathology versus repair. Defining how ILC precursor differentiation, crosstalk and plasticity contribute to disease progression, remission, and therapy resistance will be key to advancing precision immunotherapy that balances protective and pathological ILC responses (162).

## 3 ILCs in cancer

Cancer represents a complex and dynamic ecosystem comprised of cancer cells and multitudes of non-cancerous cells. The tumor microenvironment (TME) consists of stromal cells, endothelial cells, diverse immune cell populations, the extracellular matrix (ECM) and various signaling molecules such as cytokines and growth factors. Far from being a passive backdrop, the TME is continually evolving and actively influences tumor initiation, progression, metastasis and response to therapy. Its cellular composition and functional characteristics can differ extensively depending on the tissue of origin, intrinsic features of cancer cells, tumor stage, and patient-specific characteristics (163).

Within this intricate microenvironment, ILCs serve as important tissue-resident lymphocytes that provide immune surveillance within tumors by sensing cytokines and alarmins through a diverse array of surface receptors. As first responders in the TME, they secrete inflammatory or tissue-protective factors that can influence tumor progression. While traditionally classified into NK cells, ILC1, ILC2, and ILC3 subsets, recent single-cell transcriptomic analyses and comprehensive reviews have revealed considerable heterogeneity and the presence of transitional or intermediate phenotypes among these groups (8, 164–167). Reports from murine models suggest that under specific cytokine cues (e.g., TGF-β, IL-12, IL-23), ILCs may undergo functional reprogramming or phenotypic shifts — such as conversion of cytotoxic NK cells into less cytotoxic ILC1-like states, or transitions between ILC1 and IL-17-producing ILC3-like phenotypes (168–170). However, the extent to which such plasticity occurs in human tumors remains incompletely understood and alternative mechanisms — including recruitment of distinct precursors, differential expansion or contraction of subsets, and *in situ* differentiation — may also contribute to observed heterogeneity. Compared to innate-like T cells (ILTCs), which exhibit more rigid lineage commitments, ILCs appear to retain greater adaptability, positioning them as key immunological players whose context-dependent responses could potentially be therapeutically harnessed or targeted in cancer. Tumor-derived signals and inflammatory cytokines may activate antitumor programs in ILCs; conversely, TME-derived cues can polarize ILCs toward tumor-promoting phenotypes, enhancing tissue repair and homeostatic pathways. Such context-dependent alterations in ILC function have been reported across various cancers, including hepatocellular carcinoma, squamous cell carcinoma, melanoma, and colorectal cancer (8, 25, 165). The duality of ILC functions underscores the nuanced immunological landscape they govern within the TME and highlights the critical need to elucidate the molecular and cellular mechanisms shaping their roles in cancer progression and enhanced tools for evaluating true lineage plasticity versus rapid ILC subset expansion, ILC precursor differentiation *in situ*, or recruitment of distinct ILC subsets from peripheral sites. A deeper understanding of this network is essential for the rational design of effective cancer immunotherapies (**Table 2**).

**Table 2:** Functional roles of ILC subsets across different cancer types.

Innate lymphoid cells (ILCs) in cancer			
Cancer type	ILC1	ILC2	ILC3
Colorectal Cancer (CRC)	Participate in early immune surveillance through DC and T cell activation but become exhausted in advanced disease with reduced IFN-γ production (166, 171–173)	IL-25 signaling promote tumor-supportive M2 macrophages and MDSCs; IL-33 activation promotes antitumor immunity via eosinophil recruitment and T cell cooperation (174–177)	NCR^+^ subset promote TLS formation and immune recruitment (12, 178–181) NCR^-^ ILC3s produce IL-22 and IL-17, promote tumor growth and inflammation. ILC3-ILCreg conversion contributes to immune evasion (83, 108, 178, 182–187)
Melanoma	Tissue-resident ILC1s limit metastatic spread; cytotoxic functions modulated by metabolic signals (168–170)	IL-33–activated ILC2s promote antitumor immunity via eosinophil recruitment and T cell activation (159, 160, 188, 189)	Promote antitumor immunity via TRAIL-mediated cytotoxicity and immune cell recruitment through TLS formation; can contribute to metastasis via CCL21-driven immunosuppressive microenvironments, recruit immunosuppressive Tregs, and MDSCs (23, 159, 190, 191)
Pancreatic Ductal Adenocarcinoma (PDAC)	Not Known	IL-33–activated ILC2s coordinate with CD8^+^ T cells, promote TLS formation, and enhance antitumor immunity (159, 160, 188, 189)	NCR^-^ ILC3s promote tumor growth via IL-22; may contribute to immunosuppression and metastasis (178, 184, 187)
Acute Myeloid Leukemia (AML)	Not Known	IL-33–activated ILC2s exert direct cytotoxicity against AML cells via granzyme B secretion (192, 193)	Not Known
Glioblastoma (GBM) / Glioma	NK-to-ILC1 conversion driven by TGF-β may reduce cytotoxicity and promote immune evasion (194, 195)	IL-33–activation may promote eosinophil recruitment or support Th2-biased responses; Ex vivo expanded ILC2s show cytotoxicity against glioblastoma (196)	Contribute to angiogenesis and immune suppression through IL-17 and IL-22; may transition to ILCregs promoting tolerance (83, 185, 186)
Non-Small Cell Lung Cancer (NSCLC)	Exhibit cytotoxic potential early in disease; later become suppressed or exhausted (172, 173)	IL-25–activated ILC2s promote tumor progression via M2 macrophage and MDSC recruitment (174, 176, 177)	NCR^+^ ILC3s support TLS formation and recruit immune effector cells (12, 178–181) NCR^-^ ILC3s promote IL-17A–driven inflammation and tumor progression (178, 184, 187)
Hepatocellular Carcinoma (HCC)	Tissue-resident ILC1s limit metastasis; cytotoxicity modulated by local signals (168)	KLRG1^-^ ILC2s produce IL-13, CXCL2 and CXCL8, contribute to neutrophil recruitment and ARG1 activation (159, 160) KLRG1^+^ ILC2s produce IL-10 suppress T cells and contribute to immunosuppressive TME (159, 160)	Directly kill tumor cells through TRAIL-mediated cytotoxicity (23) NCR^-^ ILC3s promote tumor growth via IL-17 and IL-22; may support metastasis and immunosuppression (178, 184)

Protective and pathogenic functions of helper-type innate lymphoid cells (ILC1s, ILC2s, and ILC3s) in cancers including CRC, colorectal cancer; melanoma, PDAC, pancreatic ductal adenocarcinoma; AML, acute myeloid leukemia; GBM, glioblastoma/glioma; NSCLC, non-small cell lung cancer; HCC, hepatocellular carcinoma.

### 3.1 ILC1: early enforcers of immune surveillance and antitumor immunity

**Figure 1:**
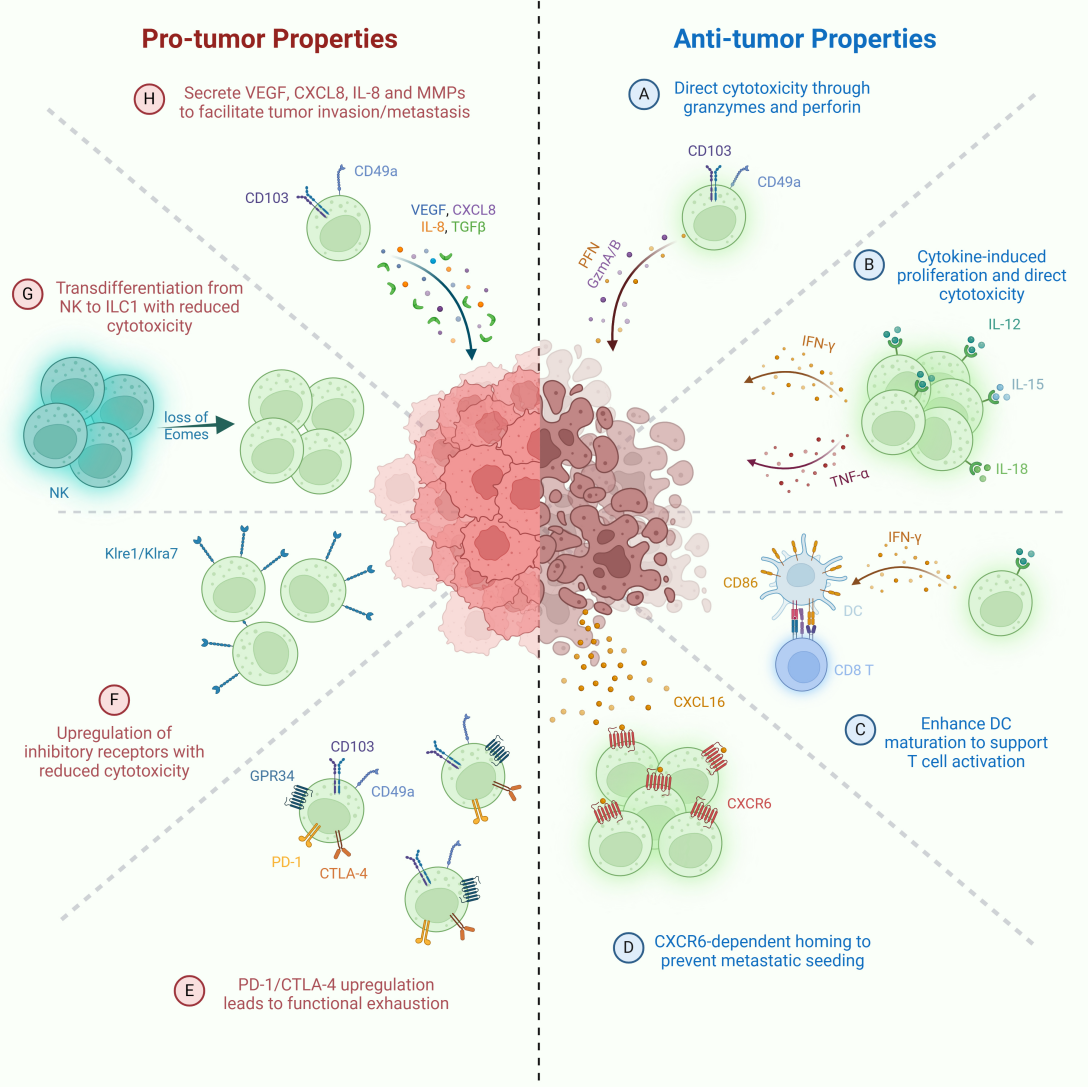
Pro-tumor and anti-tumor functions of ILC1s. Anti-tumor mechanisms: **(A)** Direct cytotoxicity against tumor cells via granzyme- and perforin-mediated killing. **(B)** IL-15-activated ILC1s secrete IFN-γ, promoting CD86 expression and DC maturation, thereby enhancing T cell priming. **(C)** Stimulation by IL-12, IL-15, and IL-18 drives ILC1 proliferation and secretion of IFN-γ and TNF, contributing to tumor cell killing. **(D)** CXCR6^+^ ILC1s migrate to CXCL16^+^ tumor sites, limiting metastatic seeding of tumor cells. Pro-tumor mechanisms: **(E)** Loss of cytotoxicity through transdifferentiation of NK cells into ILC1-like cells following downregulation of Eomes expression. **(F)** Upregulation of inhibitory receptors (e.g., Klre1, Klra7) resulting in diminished cytotoxic function. **(G)** Promotion of tumor progression via TGF-β production by CD49a^+^CD103^+^ ILC1s. **(H)** Functional exhaustion of ILC1s characterized by elevated expression of immune checkpoint molecules such as PD-1 and CTLA-4.

Group 1 innate lymphoid cells (ILC1s), once thought to be weakly cytotoxic and limited in their antitumor capacity compared to conventional NK cells, are now increasingly recognized for their potential dual roles in cancer immunity. Several recent studies have demonstrated that ILC1s can acquire cytotoxic features, producing granzyme B (GzmB), granzyme C (GzmC), perforin (PFN), IFN-γ, and TNF in response to cues from the TME such as IL-15, IL-12, and TGF-β (159, 160). ILC1s produce cytokines and cytotoxic molecules to suppress early-stage tumors via apoptotic lysis of tumor cells. In pre-cancerous mammary lesions, IL-15 secreted by tumor cells was shown to induce GzmB production in CD103^+^ cytotoxic ILC1s, contributing to early tumor surveillance (**Figure 1A**) (171). Similarly, in chromophobe renal cell carcinoma (chRCC), high infiltration of granzyme A (GzmA)-expressing intraepithelial ILC1s was positively correlated with patient survival, with IL-15 promoting both their expansion and cytotoxicity (**Figure 1A**) (172). IL-15 was also observed to boost proliferation and effector function (e.g., TNF, IFN-γ production) of ILC1s in head and neck squamous cell carcinoma, emphasizing their potential in immunosurveillance, particularly within epithelial malignancies (**Figure 1B**) (171).

In addition to their cytotoxic roles, ILC1s contribute to antitumor immunity by shaping adaptive responses. In CRC, ILC1s have been shown to activate DCs and T lymphocytes, in part through the upregulation of costimulatory molecules such as CD86, thereby promoting the development of tumor-specific immunity (**Figure 1C**). Immune cells stimulated by ILC1s demonstrate enhanced tumor infiltration, intratumoral effector activity, and are associated with reduced tumor burden (171).

Further reinforcing the tumor-suppressive potential of ILC1s, studies have identified their involvement in limiting metastatic spread. In a murine model of hepatic metastasis, tissue-resident ILC1s expressing CXCR6 localized to the metastatic site, where they were implicated in constraining tumor dissemination (**Figure 1D**) (173). However, their activity is modulated by local metabolic signals. A recent study identified G-protein-coupled receptor 34 (GPR34) as a key immune checkpoint receptor on ILC1s that senses lysophosphatidylserine (LysoPS), a lipid enriched in the TME, primarily derived from tumor cells and infiltrating apoptotic immune cells. LysoPS binding via GPR34 suppressed ILC1 activation via the cAMP-PKA-CREB signaling pathway, while genetic or pharmacological targeting of the LysoPS–GPR34 axis enhanced their antitumor functions. In both human tumors and preclinical models, elevated expression of ABHD16A (LysoPS synthase that converts cell-surface phosphatidylserine into LysoPS) or GPR34 was inversely correlated with ILC1 activity, highlighting a novel metabolic checkpoint that could be applied therapeutically to unlock ILC1-mediated tumor suppression (**Figure 1E**) (188, 189).

Despite these promising findings, ILC1s also exhibit signs of functional exhaustion and phenotypic plasticity during tumor progression, especially in CRC. In early-stage CRC, ILC1s expressed high levels of activating receptors and responded robustly to IL-12/IL-18 stimulation with IFN-γ production. However, as tumors advanced, ILC1s began to express higher levels of inhibitory receptors (e.g., Klre1, Klra7), and their ability to produce IFN-γ significantly declined (**Figure 1F**). These changes were accompanied by downregulation of *Il12rb2*, indicating impaired responsiveness to IL-12, a critical cytokine for sustaining antitumor ILC1 functions. Similarly, in advanced CRC patients, tumor-infiltrating ILC1s were found at lower frequencies and exhibited a phenotype suggestive of exhaustion, with high expression of inhibitory markers (**Figure 1E**) (197).

In addition to immune exhaustion, ILC1s can acquire pro-tumorigenic properties through phenotypic conversion and altered secretory functions. In TGF-β–rich TMEs, NK cells undergo conversion into CD49a^+^ CD103^+^ ILC1-like cells with diminished cytotoxicity and increased production of pro-angiogenic and immunosuppressive factors (168, 174). These reprogrammed ILC1-like cells contribute to immune evasion, tumor vascularization, and matrix remodeling (**Figure 1G**). Moreover, ILC1s themselves have been shown to secrete vascular endothelial growth factor (VEGF), CXCL8/IL-8, and matrix metalloproteinases (MMPs) in several cancer types, including HCC, CRC, and lung cancer, further promoting angiogenesis and tumor progression (**Figure 1H**) (168, 174).

Altogether, these studies underscore the context-dependent role of ILC1s in cancer. While capable of mounting cytotoxic responses and contributing to early immunosurveillance, their function can be subverted by tumor-intrinsic mechanisms and immunosuppressive signals in the TME. The ability to reinvigorate ILC1s—through blockade of inhibitory receptors, restoration of IL-12 signaling, or metabolic checkpoint modulation (e.g., GPR34 antagonism)—represents a promising avenue for cancer immunotherapy. However, challenges remain in selectively targeting and sustaining their beneficial antitumor properties while avoiding potential pro-tumor roles, particularly in settings where chronic stimulation may drive exhaustion or functional suppression.

### 3.2 ILC2: immune modulators in tumor progression and control

**Figure 2:**
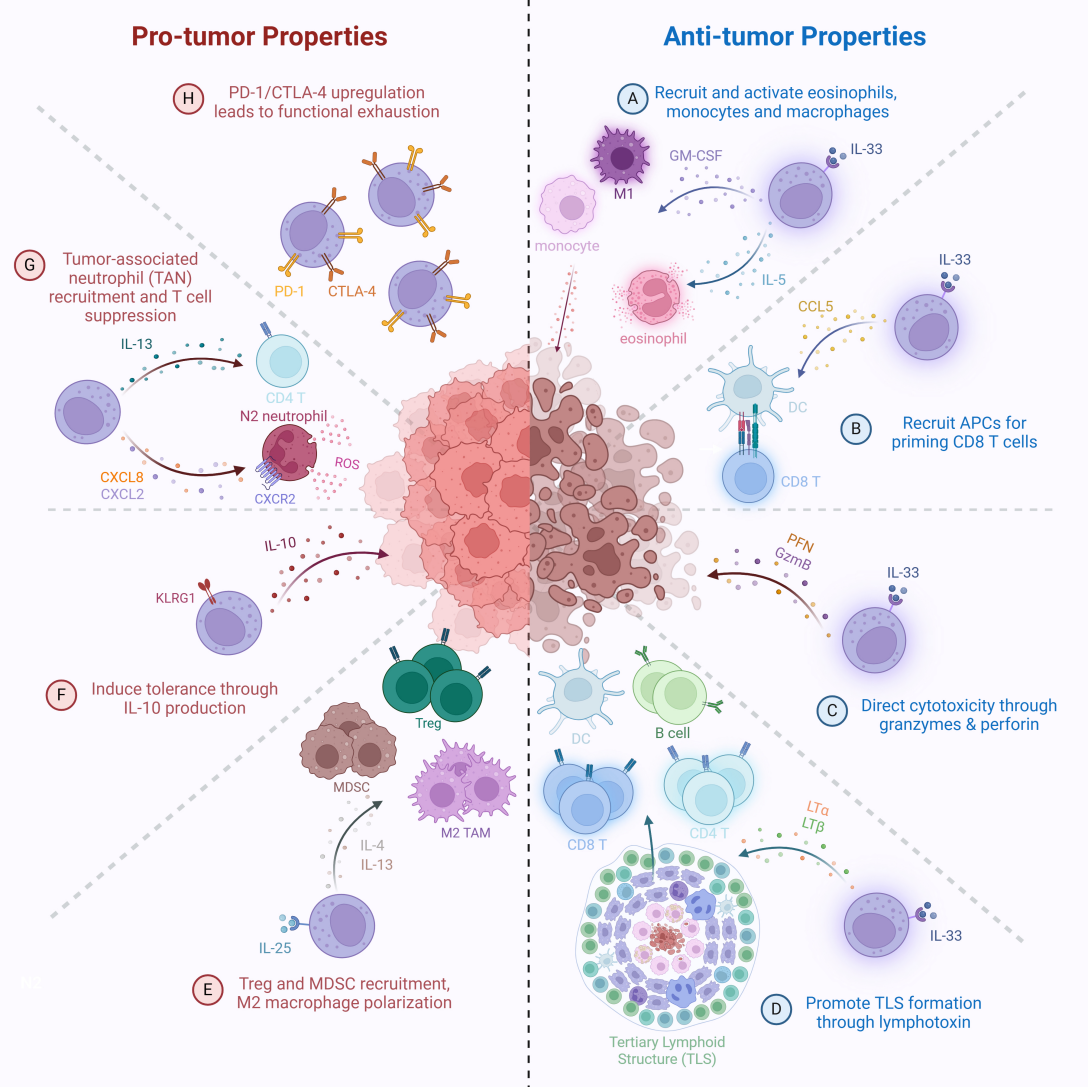
Pro-tumor and anti-tumor functions of ILC2s. Anti-tumor mechanisms: **(A)** IL-33-activated ILC2s secrete GM-CSF and IL-5 to recruit monocytes, M1-like inflammatory macrophages, and activate eosinophils for tumor cell killing. **(B)** IL-33-activated ILC2s secrete CCL5, promoting recruitment of antigen-presenting cells (APCs), such as DCs, to enhance tumor antigen presentation and prime CD8^+^ T cell responses. **(C)** Direct cytotoxicity of tumor cells via granzyme- and perforin-mediated killing. **(D)** IL-33-activated ILC2s produce lymphotoxins (LTα, LTβ), promoting tertiary lymphoid structure (TLS) formation and enhancing immune infiltration at tumor sites. Pro-tumor mechanisms: **(E)** IL-25-activated ILC2s promote recruitment of regulatory T cells (Tregs), myeloid-derived suppressor cells (MDSCs), and M2-like reparative tumor-associated macrophages (TAMs) via IL-4 and IL-13 secretion. **(F)** Induction of a tolerogenic tumor microenvironment through IL-10 production. **(G)** Recruitment of tumor-associated neutrophils (TANs) through CXCL8 and CXCL2 production, and promotion of Th2 polarization in CD4^+^ T cells, leading to suppression of anti-tumor T cell responses. **(H)** Functional exhaustion of ILC2s through upregulation of immune checkpoint molecules, including PD-1 and CTLA-4.

Group 2 innate lymphoid cells (ILC2s) have emerged as versatile regulators in cancer immunity, exhibiting both pro-tumorigenic and anti-tumorigenic functions depending on the cytokine environment, tumor type, immune context, and ILC2 subset composition (164), This duality is rooted in their plasticity and their ability to rapidly respond to epithelial-derived alarmins such as interleukin-33 (IL-33) and interleukin-25 (IL-25). On the anti-tumor side, ILC2s contribute to immune surveillance through multiple mechanisms. In melanoma, pancreatic ductal adenocarcinoma (PDAC), and other solid tumors, high levels of tumor-infiltrating ILC2s (TILC2s) correlate with favorable prognosis (175, 192). These ILC2s produce granulocyte-macrophage colony-stimulating factor (GM-CSF) and interleukin-5 (IL-5), promoting the recruitment and activation of eosinophils, which have cytotoxic and tumor-suppressive functions (**Figure 2A**) (164, 165, 192). In addition, ILC2s can interact with DCs and macrophages through GM-CSF, IL-9, and CD40L-mediated signals, enhancing antigen presentation and co-stimulatory molecule expression, which in turn promotes the priming and activation of cytotoxic T cell responses within the tumor microenvironment (**Figure 2B**) (175).

IL-33, which is secreted not only by epithelial and endothelial cells but also by damaged or necrotic tumor cells, plays a central role in this process by activating ILC2s and reshaping the TME (196). Recent findings further reveal that ILC2s help bridge innate and adaptive immunity: in immunodeficient mouse models, spleen-resident ILC2s facilitated APCs in cross-priming CD8^+^ tumor-infiltrating T cells and central memory B cells, suggesting ILC2s also influence longer-term immune memory (**Figure 2B**) (175). Mechanistically, IL-33 acts through the ST2 receptor on ILC2s, upregulating GATA3 and PD-1, the latter of which imposes a brake on their proliferation and effector function (176, 177). This inhibition can be reversed by PD-1 blockade, which synergizes with IL-33 to expand ILC2s, amplify their Th2 cytokine production, and enhance anti-tumor activity (177).

Importantly, PPARγ (peroxisome proliferator-activated receptor gamma) has been identified as a transcriptional regulator that controls PD-1 expression by ILC2s (164, 165). PPARγ is highly expressed in ILC2s compared to other ILC subsets, and its activity appears to fine-tune their functional state. In mouse models of CRC, deletion of PPARγ in ILC2s led to reduced PD-1 expression, increased anti-tumor immune responses, and better tumor control (198). These findings suggest that modulating PPARγ activity could offer an additional approach to either suppress or potentiate ILC2-mediated immune functions, depending on the therapeutic goal.

ILC2s have also demonstrated direct cytotoxicity in both hematologic and solid tumor models. *Ex vivo* expanded human ILC2s (Ex ILC2s) can kill tumor cells through the secretion of GzmB, mediated by the DNAM-1–CD112/CD155 receptor-ligand interaction (**Figure 2C**) (199). This interaction not only induces apoptosis and pyroptosis in acute myeloid leukemia (AML) cell lines and patient-derived blasts but also leads to tumor regression in mouse models of glioblastoma (178), pancreatic, and lung cancer. Importantly, DNAM-1 signaling inactivates FOXO1, a transcriptional repressor of GZMB, allowing ILC2s to gain a potent effector phenotype. These Ex ILC2s did not induce cytokine release syndrome or autoimmunity in preclinical models, indicating a favorable safety profile. Given the logistical and clinical limitations of autologous CAR-T cell therapy, especially in high-risk AML patients, off-the-shelf allogeneic Ex ILC2s, with or without CAR engineering, represent a promising and scalable immunotherapeutic platform (199).

In PDAC, ILC2s demonstrate a particularly diverse set of anti-tumor functions. IL-33–activated ILC2s not only coordinate with cytotoxic T cells to limit tumor growth (175), but also promote the formation of tertiary lymphoid structures (TLS) through lymphotoxin (LTα and LTβ) expression. These TLS, which are associated with better prognosis and enhanced T cell infiltration, form through cooperation between LT-expressing ILC2s and LTβR^+^ myeloid cells, and are influenced by the gut microbiota, which partly drives ILC2 migration from the intestine to the tumor (**Figure 2D**) (192). This highlights the tissue-specific migration and adaptation of ILC2s and their ability to act as immune organizers within solid tumors.

Conversely, ILC2s also contribute to tumor progression in multiple contexts. Under IL-25 stimulation, ILC2s adopt a pro-tumorigenic phenotype, secreting IL-4 and IL-13, which promote M2 macrophage polarization, recruit Tregs, and enhance monocytic myeloid-derived suppressor cell (MDSC) accumulation, all of which suppress cytotoxic T cell responses and enable tumor immune evasion (**Figure 2E**) (164, 165, 177, 179). In non-small cell lung cancer (NSCLC) and CRC, increased IL-25 expression correlates with worse patient survival and increased intra-tumoral ILC2 and MDSC frequencies (177, 179, 180). Adoptive transfer of IL-25-activated ILC2s into NSCLC-bearing mice results in increased tumor burden, metastasis, and reduced survival, confirming their pathogenic role (180). Blocking IL-25 signaling reverses these effects, reducing ILC2 and MDSC infiltration and restoring IFN-γ–producing CD8^+^ T cells (179). These findings demonstrate the immunosuppressive capabilities of IL-25–driven ILC2s and point to IL-25 signaling as a therapeutic target in specific tumor types.

A critical factor in understanding these opposing roles is the subset heterogeneity of ILC2s. Subsets defined by KLRG1 expression appear to have distinct functions: KLRG1^+^ ILC2s can become regulatory, producing IL-10 and inducing tolerance (**Figure 2F**), while KLRG1^-^ ILC2s are associated with IL-13, CXCL2, and CXCL8 production and neutrophil recruitment, contributing to ARG1-mediated T cell suppression in hepatocellular carcinoma (HCC) (**Figure 2G**) (164, 165). Similarly, LKB1-deficient ILC2s in lung cancer show elevated PD-1 expression due to activation of the NFAT pathway, leading to functional exhaustion (**Figure 2H**) (181). However, PD-1 blockade restores their effector functions, highlighting the interplay between metabolic regulation and immune checkpoint control in modulating ILC2 activity (181, 190).

In sum, ILC2s function as critical immune modulators with dualistic roles in cancer. Their pro-tumor activities can be curtailed by targeting cytokine signals (like IL-25) or reprogramming their inhibitory checkpoints (like PD-1), while their anti-tumor capacities can be harnessed by combining IL-33-based activation with checkpoint inhibitors. A deeper understanding of ILC2 subsets, plasticity, and interactions with other immune cells will be essential for developing ILC2-targeted therapies tailored to specific tumor environments.

### 3.3 ILC3: context-dependent guardians or enablers in cancer

**Figure 3:**
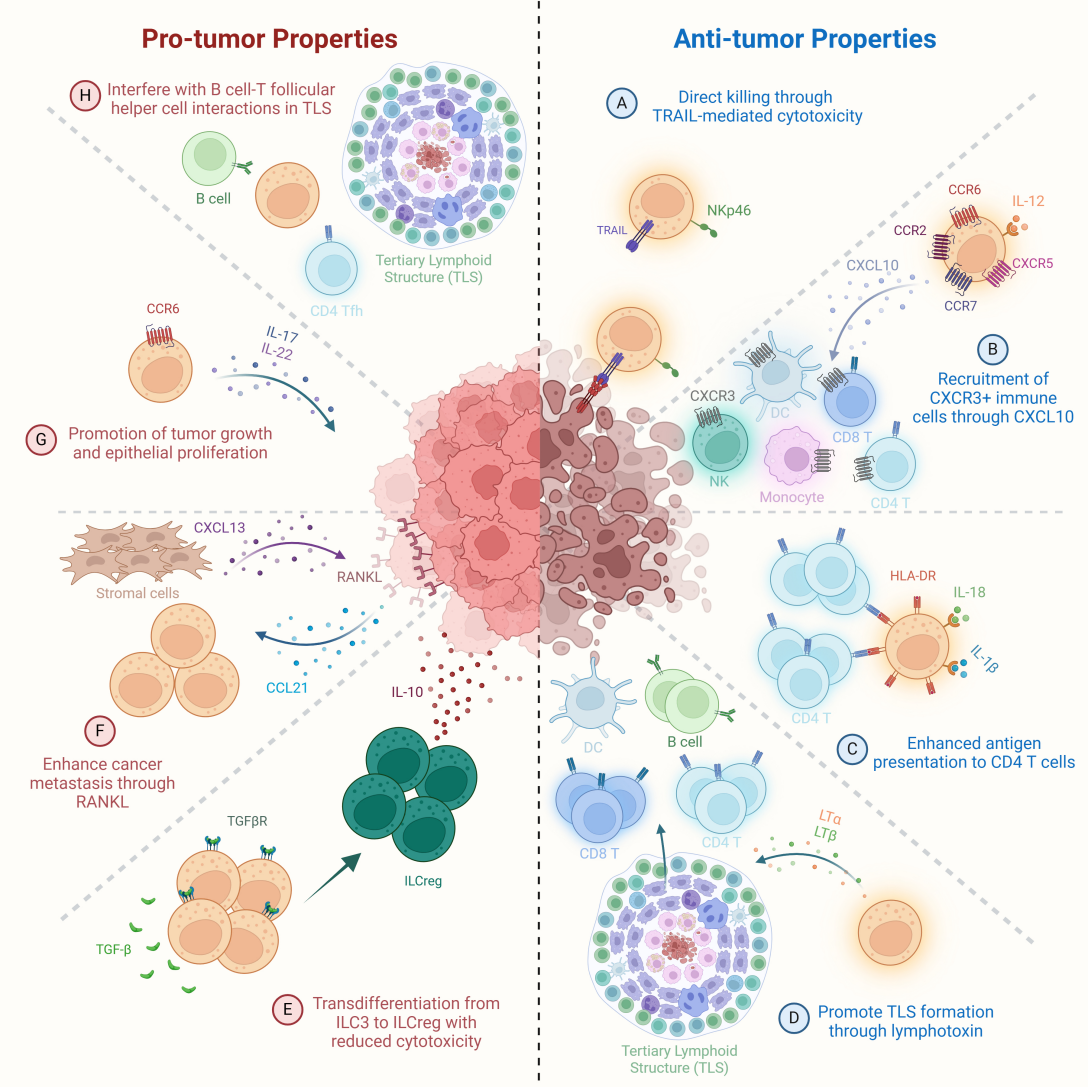
Pro-tumor and anti-tumor functions of ILC3s. Anti-tumor mechanisms: **(A)** Direct cytotoxicity of tumor cells by NKp46^+^ ILC3s via TRAIL-mediated killing. **(B)** IL-12-stimulated ILC3s secrete CXCL10, promoting recruitment of CXCR3^+^ NK cells, monocytes, DCs, and CD4^+^ and CD8^+^ T cells to enhance immune infiltration within the tumor microenvironment. **(C)** IL-18 and IL-1β-activated ILC3s upregulate HLA-DR expression, enhancing antigen presentation to CD4^+^ T cells. **(D)** Secretion of lymphotoxins (LTα, LTβ) by ILC3s promotes tertiary lymphoid structure (TLS) formation and supports anti-tumor immune surveillance. Pro-tumor mechanisms: **(E)** TGF-β in the tumor microenvironment drives transdifferentiation of ILC3s into regulatory ILCs (ILC_reg_), reducing cytotoxicity and promoting immune tolerance. **(F)** Tumor cell-derived CCL21 recruits ILC3s to the tumor microenvironment, where they induce CXCL13 secretion by stromal cells, leading to RANKL upregulation on tumor cells and enhanced metastatic potential. **(G)** CCR6^+^ ILC3s secrete IL-17 and IL-22, promoting tumor growth and epithelial proliferation. **(H)** ILC3s disrupt B cell–T follicular helper (T_FH_) cell interactions within TLSs, impairing local immune activation and anti-tumor surveillance.

Group 3 innate lymphoid cells (ILC3s), like ILC2s, represent a highly heterogeneous population whose roles in cancer range from tumor-suppressive to tumor-promoting, depending on their subset, cytokine production, and the tumor microenvironment (TME). Anti-tumor functions of ILC3s have been demonstrated in several cancer models. *In vitro*, ILC3s can directly kill hepatocellular carcinoma and melanoma cells via TRAIL-mediated cytotoxicity following tumor cell recognition through NKp46 (**Figure 3A**) (25). *In vivo*, NKp46^+^ ILC3s in non-small cell lung cancer (NSCLC) did not exhibit direct cytotoxicity but responded to tumors by producing IL-8 and TNF, and localized near tertiary lymphoid structures (TLS), which are associated with favorable prognosis (14). In both NSCLC and early-stage colorectal cancer (CRC), higher densities of NCR^+^ ILC3s correlated with increased TLS formation and elevated expression of LTα, LTβ, and TNF, suggesting that ILC3s contribute to shaping immune-supportive niches within the TME (**Figure 3D**) (14, 182, 194, 200, 201).

ILC3s also enhance anti-tumor immunity by recruiting immune effector cells. CCR6^+^ ILC3s in the spleen, when stimulated with IL-12, upregulate chemokine receptors such as CCR2, CCR6, CCR7, and CXCR5, leading to increased infiltration of CD8^+^ T cells, NK cells, NKT cells, and myeloid cells into B16 melanoma tumors (164, 183). In lung cancer models, CCR6^+^ ILC3s producing CXCL10 attracted CXCR3^+^ immune cells—NK cells, T cells, DCs, and monocytes—slowing tumor progression (**Figure 3B**) (202). Moreover, chemotherapeutic agents like cisplatin appear to enhance ILC3 function. In a mouse lung cancer model, cisplatin elevated tumor IL-1β and CCL20 levels, recruiting and activating ILC3s to produce CXCL10 and bolster antitumor immunity (184).

ILC3s also participate in adaptive immune regulation. Upon stimulation with IL-1β and IL-18, human ILC3s upregulate HLA-DR and co-stimulatory molecules in an NF-κB-dependent manner, promoting CD4^+^ T cell activation (**Figure 3C**) (203). This enhanced antigen presentation leads to increased proliferation of tumor−specific CD4^+^ T cells and upregulation of their effector cytokines, particularly IFN−γ and IL−2, thereby potentiating antitumor immunity (159, 160, 200, 201). However, TGF-β, a cytokine abundantly expressed in many tumors, can suppress this antigen-presenting capacity. In the gut, ILC3s expressing MHCII interact with CD4^+^ T cells to support commensal colonization and type 1 immunity. Loss of MHCII expression on ILC3s in mice results in spontaneous CRC development and resistance to anti-PD-1 therapy, highlighting their potential as immunotherapeutic allies (83, 191). These findings suggest that ILC3s can bridge innate and adaptive immunity by activating CD4+ T cells, thereby converting ‘cold’ tumors into ‘hot’ ones and enhancing checkpoint blockade responsiveness.

Conversely, ILC3s can adopt tumor-promoting roles. In a *Helicobacter hepaticus*–driven CRC model, CCR6^+^ ILC3s were the primary source of IL-22, which promoted epithelial proliferation and tumorigenesis (**Figure 3G**) (151). Correspondingly, DC-derived IL-22 binding protein (IL-22BP) can inhibit this effect, reducing proliferation in the colon (185). ILC3s have also been implicated in promoting metastasis. In breast cancer, increased ILC3 presence correlates with lymph node metastasis and poor prognosis (186). In mouse models, tumor-derived CCL21 recruits NKp46^-^ ILC3s, which trigger stromal CXCL13 production, leading to RANKL expression and enhanced cancer cell motility and dissemination (**Figure 3F**) (187). Similar mechanisms are observed in melanoma, where CCL21 mediates recruitment of NKp44^-^ ILC3s, regulatory T cells (Tregs), and MDSCs, establishing a tolerogenic microenvironment (204).

Another axis of ILC3-driven tumor support is their plasticity. In advanced CRC, ILC3s undergo transdifferentiation into IL-10–secreting regulatory ILCs (ILCregs) under the influence of TGF-β. These ex-ILC3s lose RORγt expression and adopt a suppressive phenotype, facilitating immune evasion (**Figure 3E**) (191, 205). Single-cell RNA-sequencing and lineage tracing confirm this transition, revealing the tumor-stage specificity of this switch. Blocking ILC3-to-ILCreg conversion reduces tumor burden in mouse models, suggesting a novel immunotherapeutic angle for CRC (206).

The duality of ILC3 function is mirrored in their cytokine outputs. NCR^+^ ILC3s tend to be anti-tumoral, producing IL-22 and TNF in contexts such as early-stage lung cancer and mucosal immunity, whereas NCR^-^ ILC3s typically produce IL-17 and support tumor growth in CRC, liver, and pancreatic cancers (186, 194). Additionally, ILC3s may interfere with B cell–T follicular helper (Tfh) cell interactions in TLS, suppressing IgA responses and potentially weakening humoral anti-tumor immunity (**Figure 3H**) (194, 207).

ILC3s play a multifaceted role in cancer immunity, with anti-tumor effects primarily driven by their ability to recruit and activate effector cells, produce cytokines like IL-22 and TNF, form TLS, and present antigens. However, under certain conditions—such as exposure to IL-23, TGF-β, or tumor-derived chemokines—ILC3s can promote tumor growth, metastasis, and immune suppression. This functional plasticity highlights the need for careful characterization of ILC3 subsets within specific tumor contexts to harness their full therapeutic potential.

## 4 Discussion: advancing ILC-based therapies by navigating the pitfalls and promise

ILCs represent a promising frontier in the development of immunotherapies for cancer and autoimmune diseases. Recent research has uncovered their capacity to exert potent regulatory or cytotoxic functions in various contexts, leading to a surge in interest in their therapeutic potential. In general, among their subsets, ILC1s are largely cytotoxic and pro-inflammatory, ILC2s are immunomodulatory and tissue-protective, while ILC3s participate in barrier defense but can shift toward immunosuppression under certain cues. Unlike T or NK cells, ILCs adapt quickly to local microenvironments, making them attractive targets for precision immunotherapy.

### 4.1 Subset-specific therapeutic roles

Recent studies have uncovered potent regulatory and cytotoxic roles for ILCs in various pathologies, catalyzing efforts to harness them therapeutically. For example, ILC2s producing IL-10 (ILC2_10_), have demonstrated robust immunomodulatory functions in the context of graft-versus-host disease (GVHD). Reid et al. and Colpitts et al. showed that these cells suppress pathogenic CD4^+^ and CD8^+^ T cell responses through IL-10 and IL-4 production, reducing Th1/Tc1 polarization, limiting tissue infiltration, and attenuating cytotoxicity. In GVHD models, ILC2_10_ cells decreased intestinal T cell infiltration and inflammation while preserving graft-versus-leukemia (GVL) activity, and higher circulating ILC2_10_ frequencies in patients correlated with lower GVHD incidence without increased relapse risk (193, 208). Beyond GVHD, ILC2_10_ cells hold promise for treating autoimmune and inflammatory conditions such as multiple sclerosis, type 1 diabetes, systemic lupus erythematosus, and inflammatory bowel disease, where excessive T cell-mediated inflammation is central to pathology. Additionally, their regulatory phenotype may offer therapeutic benefits in type 2-driven diseases like severe asthma and atopic dermatitis by modulating immune imbalance without broadly suppressing immunity. This cytokine-based, non-cytolytic mode of action distinguishes ILC2_10_ cells as a versatile platform for cell-based immunotherapies.

In parallel, ILC1s are being recognized for their capacity to mediate direct tumor cell killing via interferon-gamma (IFN-γ) production. Verner et al. highlight that ILC1s exhibit cytotoxicity against cancer cells and can be pre-activated with IL-12 or IL-15 to enhance their anti-tumor efficacy. However, ILC1s are vulnerable to immunosuppressive signals within the tumor microenvironment (TME), particularly from cytokines like TGF-β and IL-23, which in some cases has been reported to reprogram them into pro-tumoral ILC3-like cells (209).

### 4.2 Engineering ILCs to overcome limitations

Engineering approaches such as chimeric antigen receptors (CARs) and chimeric switch receptors (CSRs) are emerging as promising tools to overcome key limitations of ILCs—namely, their lack of inherent antigen specificity and susceptibility to functional plasticity within suppressive TMEs. CARs are synthetic receptors that couple an antigen-recognition domain, typically derived from antibodies, with intracellular signaling motifs to activate immune cells upon engagement with target antigens. Originally developed for T cell-based therapies, CARs are now being applied to ILCs to confer tumor specificity and enhance anti-cancer functions. Proof-of-concept studies by Ueda et al. and Suwen Li et al. have demonstrated that iPSC-derived CAR-expressing ILC/NK-like cells can effectively target glypican-3 (GPC3) in hepatocellular carcinoma and CD19 in B-cell malignancies (210, 211). These findings establish the feasibility of scalable, allogeneic, off-the-shelf ILC-based immunotherapies.

Despite promising advances, several translational barriers remain before CAR-engineered ILCs can become clinically viable. A key concern is the risk of on-target/off-tumor toxicity, particularly in solid tumors where antigen heterogeneity and limited tumor-specificity can compromise safety and efficacy—challenges well documented in CAR-T therapies (212, 213). Moreover, the *in vivo* persistence and functionality of CAR-ILCs are not yet well understood. These cells may undergo exhaustion or be eliminated in immunosuppressive microenvironments rich in cytokines such as TGF-β and IL-10, which impair effector activity (214, 215). Although ILCs lack TCRs and do not cause graft-versus-host disease, allogeneic CAR-ILCs may still trigger host immune rejection, necessitating immune-evasive strategies such as HLA class I knockout or CD47 overexpression (216). Additionally, there is an opportunity for innovation in process development—specifically, to engineer streamlined, GMP-compliant manufacturing workflows and to optimize vector delivery systems that lower cost and technical complexity, leveraging lessons from advances in the CAR-T field (212, 213, 217).

**Figure 4:**
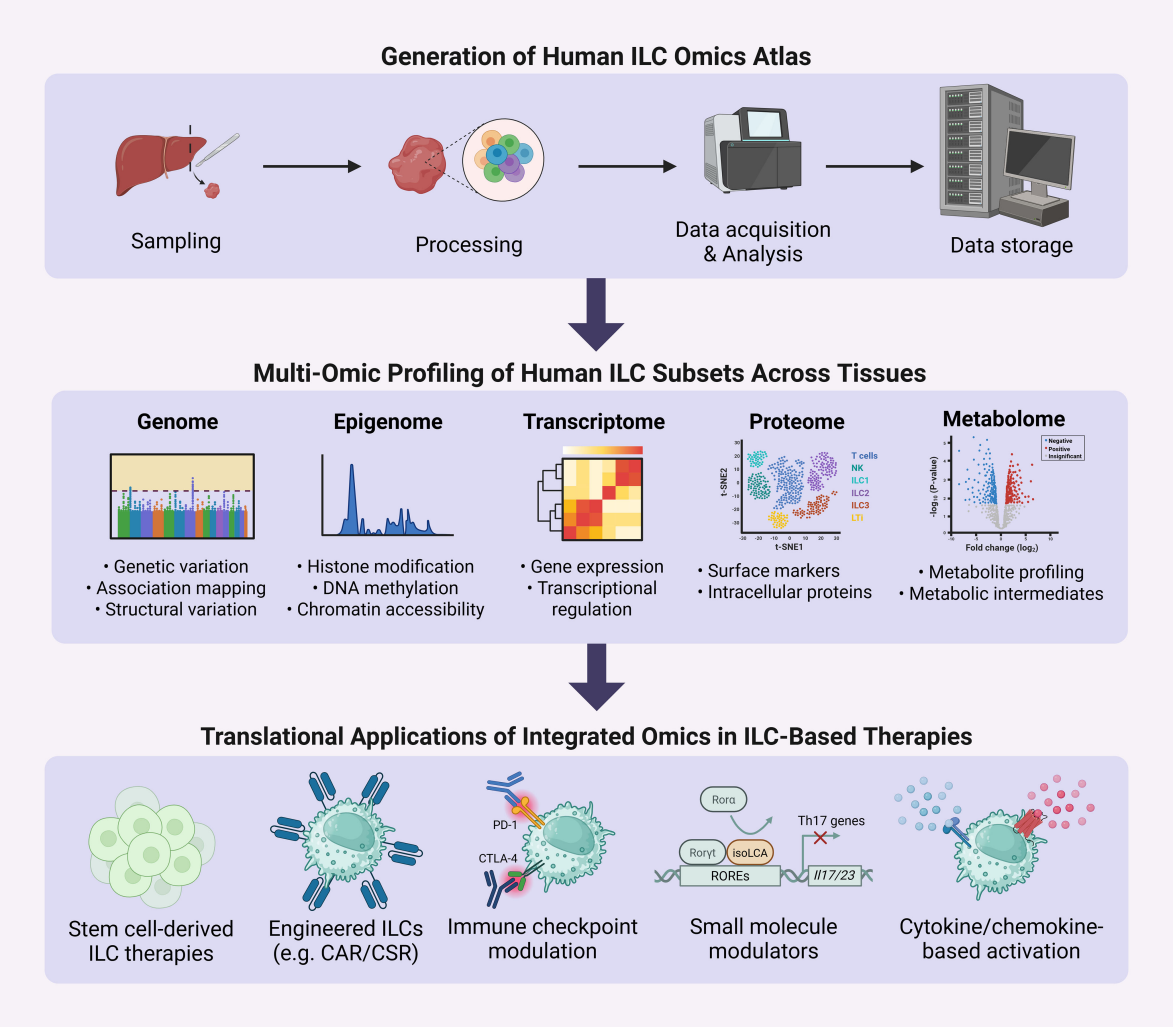
Workflow for generation of a human ILC omics atlas. Sampling of healthy and diseased human tissues enables acquisition of multi-omic datasets—including genomic, epigenomic, transcriptomic, proteomic, and metabolomic profiles—across ILC subsets. Integration of these datasets through public repositories facilitates rapid exploration of single-cell and multi-omic data, enabling analyses across species, tissues, developmental stages, and disease states. Multi-omic profiling includes genomic variation and association mapping to identify genetic variants influencing ILC development and function; epigenomic signatures such as histone modifications, DNA methylation, and chromatin accessibility to reveal the regulatory landscape controlling ILC plasticity; transcriptomic regulation and gene expression patterns to define ILC subset identities and activation states; proteomic characterization of cell surface markers and intracellular proteins to refine functional phenotypes and enable precise subset isolation; and metabolomic profiling of metabolites and metabolic intermediates to uncover the metabolic dependencies that govern ILC effector functions and persistence. This integrated approach advances understanding of ILC tissue residency, plasticity, and therapeutic potential, and informs translational strategies such as the generation of stem cell-derived ILCs for therapy, engineering of chimeric antigen or switch receptors to enhance function, modulation of immune checkpoint pathways to prevent exhaustion, application of small molecules to guide transcriptional programs, and cytokine- and chemokine-based activation of ILCs for disease modulation.

Building on CAR strategies, CSRs offer a complementary approach to overcome the immunosuppressive cues within the tumor microenvironment. CSRs are engineered receptors that fuse an extracellular inhibitory ligand-binding domain with an activating intracellular signaling domain, enabling immune cells to convert suppressive signals into stimulatory cues. Although CSR engineering in ILCs remains to be validated in peer-reviewed studies, initial concepts suggest this could be an exciting direction for future therapies. By redirecting inhibitory signals such as PD-L1 and TGF-β through synthetic receptors with activating domains (e.g., CD28, 4-1BB), CSRs could enhance the persistence and function of ILCs within immunosuppressive tumor microenvironments (TMEs). This approach may be particularly valuable for sustaining the regulatory or cytotoxic functions of ILC2s and ILC1s, respectively. Further refinement of CAR and CSR designs, incorporation of safety switches, and dual-antigen targeting strategies will be crucial to enhancing specificity, minimizing toxicity, and improving the therapeutic potential of engineered ILCs (**Figure 4**).

### 4.3 Scalable production: CB-derived ILCs

In parallel with engineering efforts, advances in scalable production platforms are expanding the feasibility of ILC-based therapies. Zhenglong Li et al. demonstrated the safety and efficacy of *ex vivo* expanded ILC2s (Ex ILC2s) in AML models, showing tumor cell killing through GzmB without inducing cytokine release syndrome or neurotoxicity (218). Unlike CAR-T cells, Ex ILC2s can be used allogeneically and administered post-remission to prevent relapse, making them promising alternatives in aggressive leukemias.

To further expand access, *ex vivo* differentiation from umbilical cord blood (CB)-derived CD34^+^ hematopoietic stem cells provide a scalable, allogeneic source for therapeutic applications (22, 195, 219–222). Specifically, stromal co-culture of Lin^-^CD34^+^α4β7^+^ progenitors, sorted by CD48/CD52, enables guided differentiation into NK, ILC1, ILC2, and ILC3 subsets using defined cytokine cocktails—2B4 (CD48-CD244) signaling influences NK versus ILC2 fate decisions (223). Further refinement of ILC3 generation has been achieved through death receptor 3 (DR3) ligation by its ligand, TL1A, which serves as a potent costimulatory signal. *In vitro*, TL1A enhances expansion and IL-22 production by ILC3s when combined with IL-1β and IL-23, even without APCs (224). This DR3/TL1A axis thus offers a means of driving both quantity and functional quality of CB-derived ILC3, with translational implications for barrier tissue repair and mucosal immunity.

### 4.4 Emerging strategies and challenges: pluripotent stem cell platforms

Despite these advances, one major bottleneck in the translation of ILC-based therapies is the limited abundance and accessibility of ILCs in human tissues. Unlike T cells and NK cells, which can be readily isolated from peripheral blood and expanded using established protocols, ILCs are rare in circulation and are primarily tissue-resident. This scarcity complicates efforts to obtain sufficient cell numbers for experimental and therapeutic use. Additionally, ethical and logistical barriers related to accessing human tissues rich in ILCs (e.g., intestinal mucosa, lung, skin) pose further challenges. These constraints accentuate the importance of exploring pluripotent stem cell (PSC) platforms, which offer a renewable, ethically viable, and scalable source for generating ILC-like cells. Current progress in ILC-based therapy design increasingly leverages PSC-derived models and xenograft systems, which enable controlled manipulation and mass production of ILCs for both research and clinical applications.

However, important challenges remain before PSC-derived ILC products can be advanced toward translational pipelines. Immunogenicity remains a concern, as reprogramming or incomplete differentiation may result in expression of non-self or fetal antigens, potentially triggering host-versus-graft responses (215). Another challenge is functional heterogeneity. Differentiation protocols often yield mixed populations with variable phenotypes, complicating reproducibility and potency assessment. Refining lineage-specific conditions, improving quality control, and establishing robust quality attributes will be critical for advancing these platforms (213). Moreover, the regulatory landscape outlining standardized criteria for identity, purity, and safety testing of PSC-derived ILC products remains to be fully defined. Robust GMP-compliant workflows and validated preclinical models will be essential to ensure safety and consistency at clinical scale (212). While the promise of PSC-derived ILCs in immunotherapy is compelling, realizing their full potential will require sustained interdisciplinary efforts to overcome current technical and regulatory challenges.

### 4.5 Mechanistic insights and translational opportunities

As the therapeutic landscape for ILCs continues to evolve, recent insights have expanded the potential of ILC-based strategies by highlighting their responsiveness to immune checkpoint blockade, their role in immunoregulatory circuits, and their plasticity under tissue-dependent cues. These approaches are not mutually exclusive, but part of a broader paradigm aimed at sustaining and directing ILC activity in the face of environmental signals.

In the realm of immune checkpoint blockade, preclinical work in pancreatic cancer models have shown that combining recombinant IL-33 with PD-1 checkpoint inhibition significantly enhances ILC2-mediated tumor control and prolongs survival, underscoring how checkpoint blockade can reinforce ILC activity within immunosuppressive microenvironments (175). Similarly, the discovery of co-regulatory interactions between ILCs and T cells in secondary lymphoid tissues suggests that molecules like CTLA-4 (225) could be manipulated to sustain this immune synergy, optimizing antigen presentation and cytokine production (226–229).

Moreover, the plasticity of ILCs opens the new possibility for transdifferentiation-based therapies. In CRC, ILC3s have been shown to transdifferentiate into immunosuppressive ILCreg cells in response to elevated TGF-β levels in the TME, a shift that coincides with advanced cancer stages (202, 230). Preventing or delaying this transition through cytokine modulation or small molecule intervention could preserve the tumor-suppressive identity of ILCs and create a temporal advantage for immune-mediated tumor clearance (231, 232). These discoveries provide critical mechanistic insights and translational opportunities to enhance the durability and specificity of ILC-driven therapies.

### 4.6 Future directions: technologies and generation of ILC omics atlas

Looking ahead, the integration of ILC-based therapies with current immunotherapeutic strategies, such as immune checkpoint inhibitors or metabolic modulators, holds considerable promise for enhancing treatment efficacy in cancer and immune-mediated diseases. Advances in gene-editing technologies, including CRISPR/Cas9, and synthetic biology offer unprecedented opportunities to engineer ILCs with precise phenotypes and functional profiles tailored to specific therapeutic needs. Additionally, bioengineered platforms—such as patient-derived organoids and organ-on-chip systems—are emerging as powerful tools to model ILC behavior in disease-relevant contexts and to test personalized interventions *ex vivo*. These innovations also facilitate the development of longitudinal studies that track ILC lineage, fate and function over time, providing critical insights into their roles in health and disease progression.

Historically, the study of ILCs has been hampered by the scarcity of tissue samples, the complexity of their phenotypic heterogeneity, and limitations in technologies. However, the advent of high-resolution single-cell technologies now enables unprecedented resolution in characterizing ILC subsets, their microenvironmental interactions, and their activation states. Techniques such as single-cell RNA sequencing, assay of transposable-accessible chromatin sequencing (ATAC-Seq) and cellular indexing of transcriptomes and epitopes by sequencing (CITE-Seq) are being employed to uncover key regulatory pathways and transcriptional programs in rare ILC populations. Spatial transcriptomics and advanced 3D imaging further allow the mapping of ILCs within their native tissue contexts, revealing how local microenvironments shape their function (233). Public platforms like CZ CELLxGENE Discover facilitate rapid exploration of published single-cell transcriptomic datasets, enabling integrative and exploratory analyses across different species, tissues, developmental stages, and diseases.

Metabolic profiling and regulation represent another key dimension of ILC biology, as metabolism governs effector plasticity, persistence, and therapeutic responsiveness in cancer and autoimmune settings. Distinct metabolic programs underlie ILC subset function: ILC1s and NK cells rely on mTOR-driven glycolysis while ILC2s utilize arginase-1–mediated polyamine synthesis to sustain glycolytic flux, and ILC3s depend on oxidative phosphorylation to support IL-17 and IL-22 production (18, 19). Dysregulated ILC metabolism contributes to immune evasion in tumors and to pathogenic activation in autoimmune diseases such as inflammatory bowel disease (IBD) and systemic lupus erythematosus (SLE). Emergent technologies are offering unprecedent insights into the ways metabolism regulate function. For example, single-cell energetic metabolism by profiling translation inhibition (SCENITH) offers a flow cytometry–based method to resolve metabolic dependencies in rare tissue-resident ILCs by quantifying protein synthesis under selective metabolic inhibition (19). Complementary spatial and proteogenomic technologies like Single-Cell Proteomics by Mass Spectrometry (SCoPE2), Multiplexed Ion Beam Imaging by Time-of-Flight (MIBI-TOF), and Spatial Metabolomics (SpaceM)—enable *in situ* mapping of ILC metabolic states within the tumor microenvironment or inflamed tissues. Leveraging these advanced tools will be essential for identifying metabolic targets to modulate ILC function and for optimizing ILC-based immunotherapies.

Together, these tools are transforming our understanding of how ILCs orchestrate immune responses within complex tissue landscapes. As these technologies mature, they will be instrumental in unraveling the immunoregulatory networks that ILCs inhabit and influence, ultimately guiding the rational design of ILC-targeted interventions across multiple disease domains (**Figure 4**).

## 5 Conclusion

The growing recognition of ILCs as versatile immunological effectors positions them as extremely promising candidates for next-generation cell therapies. Their intrinsic ability to respond rapidly to local tissue cues and mediate both protective and pathogenic responses underscore their potential to complement, or even improve upon, current cell-based therapies. Continued advancements in ILC biology, when paired with innovations in regenerative medicine and cellular engineering, could lead to safe, targeted, and flexible treatment options across a wide range of malignancies and immune-mediated diseases.
